# Advances in regenerative medicine applications of tetrahedral framework nucleic acid-based nanomaterials: an expert consensus recommendation

**DOI:** 10.1038/s41368-022-00199-9

**Published:** 2022-10-31

**Authors:** Yunfeng Lin, Qian Li, Lihua Wang, Quanyi Guo, Shuyun Liu, Shihui Zhu, Yu Sun, Yujiang Fan, Yong Sun, Haihang Li, Xudong Tian, Delun Luo, Sirong Shi

**Affiliations:** 1 State Key Laboratory of Oral Diseases & National Clinical Research Center for Oral Diseases & Department of Oral and Maxillofacial Surgery, West China Hospital of Stomatology, Sichuan University, Chengdu, China; 2 School of Chemistry and Chemical Engineering, Frontiers Science Center for Transformative Molecules, Institute of Translational Medicine, Shanghai Jiao Tong University, Shanghai, China; 3 The Interdisciplinary Research Center, Shanghai Advanced Research Institute, Chinese Academy of Sciences, Zhangjiang Laboratory, Shanghai, China; 4 Institute of Orthopedics, Chinese PLA General Hospital, Beijing Key Laboratory of Regenerative Medicine in Orthopedics, Key Laboratory of Musculoskeletal Trauma & War Injuries PLA, Beijing, China; 5 Department of Burn Surgery, The First Affiliated Hospital of Naval Medical University, Shanghai, China; 6 National Engineering Research Center for Biomaterials, Sichuan University, Chengdu, China; 7 College of Biomedical Engineering, Sichuan University, Chengdu, China; 8 Jiangsu Trautec Medical Technology Company Limited, Changzhou, China; 9 Chengdu Jingrunze Gene Technology Company Limited, Chengdu, China

**Keywords:** Biomedical engineering, Translational research

## Abstract

With the emergence of DNA nanotechnology in the 1980s, self-assembled DNA nanostructures have attracted considerable attention worldwide due to their inherent biocompatibility, unsurpassed programmability, and versatile functions. Especially promising nanostructures are tetrahedral framework nucleic acids (tFNAs), first proposed by Turberfield with the use of a one-step annealing approach. Benefiting from their various merits, such as simple synthesis, high reproducibility, structural stability, cellular internalization, tissue permeability, and editable functionality, tFNAs have been widely applied in the biomedical field as three-dimensional DNA nanomaterials. Surprisingly, tFNAs exhibit positive effects on cellular biological behaviors and tissue regeneration, which may be used to treat inflammatory and degenerative diseases. According to their intended application and carrying capacity, tFNAs could carry functional nucleic acids or therapeutic molecules through extended sequences, sticky-end hybridization, intercalation, and encapsulation based on the Watson and Crick principle. Additionally, dynamic tFNAs also have potential applications in controlled and targeted therapies. This review summarized the latest progress in pure/modified/dynamic tFNAs and demonstrated their regenerative medicine applications. These applications include promoting the regeneration of the bone, cartilage, nerve, skin, vasculature, or muscle and treating diseases such as bone defects, neurological disorders, joint-related inflammatory diseases, periodontitis, and immune diseases.

## Introduction

Deoxyribonucleic acid (DNA), which carries genetic information to synthesize RNA and proteins, is an essential biological macromolecule for the development and function of organisms.^1,2^ It is a biopolymer comprising deoxynucleotides; these building blocks contain deoxyribose, phosphate, and one of four nucleobases: thymine (T), adenine (A), guanine (G), and cytosine (C). Taking advantage of the Watson–Crick complementary base pairing, A, G, T, and C can selectively bind to each other (A-T, G-C) through reversible hydrogen bonds to form double-helical DNA structures.^3^ G and C interact via three hydrogen bonds, which is more stable than that of A and T, which are linked with only two hydrogen bonds. The programmability, predictability, and addressability of DNA make it an excellent and attractive material for biology, physics, medicine, and engineering applications. “Structural DNA nanotechnology,” once proposed by Nadrian “Ned” Seeman at the beginning of the 1980s, has opened the door to an emerging field of DNA research.^4^ As the founding father of DNA nanotechnology, Seeman suggested that DNA molecules could be used as a versatile building block for self-assembly to form a more advanced, complex, multidimensional nanostructure according to the rigid complementary base paring rules. Increasing evidence indicates that the suitable sizes and hydrophilic surfaces of self-assembled DNA nanostructures can autonomously enter mammalian cells in the absence of transfection agents.^5,6^ In addition, the ability of DNA nanostructures to resist nuclease degradation has also been greatly improved compared with single-stranded DNA (ssDNA) or double-stranded DNA (dsDNA) in physiological environments.^7–9^

Four decades of rapid development have witnessed the convenient construction of various two-dimensional (2D) and three-dimensional (3D) nanoscale DNA assemblies with unprecedented accuracy and complexity.^10–13^ Duplex or triplex-hybridization DNA nanostructures could be quickly formed according to the A-T and G-C complementary rules.^14^ Moreover, the scaffolded DNA origami led to the cross-era progress in structural DNA nanotechnology since Rothemund reported in the early 2000s.^10^ He proposed a simple method to generate arbitrary shapes of 2D nanostructures by raster-filling the targeted shape with a long single-stranded “DNA scaffold strand” and holding the scaffold in place via short single-stranded “staple strands.” Since then, the fabrication of 3D DNA nanostructures has been supported by that method, which lets the shapes twist and bend according to custom curvatures.^15,16^ Except for static DNA nanostructures such as DNA tiles,^17,18^ DNA origami,^19–21^ and spherical nucleic acids (SNAs),^22^ dynamic DNA nanostructures responding to changes in temperature,^23,24^ pH,^25–28^ metal ion concentration,^29–31^ enzymes,^32^ small molecules,^33–35^ and sequence-specific oligonucleotides^31,36–38^ could be designed to self-assemble with highly ordered and well-defined systems according to the well-known Watson–Crick base pairing principle, through the reversible nature of hydrogen bonds.

DNA possesses a variety of unique properties, which makes it easy to successfully design and construct complex, dynamic, and functional nanostructures. Because of this, self-assembled DNA nanostructures have remarkable advantages compared to other conventional nanomaterials. First, since the structural unit of these nanostructures is DNA, they essentially have low cytotoxicity, are biocompatible, biodegradable, and have a low probability of triggering an immune response, enabling them to be used in vivo and in vitro.^39–41^ Second, benefiting from the Watson-Crick complementary base pairing, DNA can efficiently and explicitly self-assemble into well-defined and uniform one-dimensional to three-dimensional DNA nanostructures according to a designed program with incredible structural diversity and complexity.^10,21,42–45^ In addition, based on the reversible hydrogen binding principle, researchers can create different DNA nanostructures with controllable structures and powerful functions, which can change their conformation in response to a variety of external stimuli such as pH, sequence-specific oligonucleotides, metal ions, enzymes, small molecules, and temperature.^46,47^ Finally, DNA nanostructures can be functionalized through modification with various molecules (including aptamers, nanoparticles, drugs, proteins, and dye molecules) on their surfaces in an accurate and controllable way.^48,49^ These excellent properties of DNA nanostructures allow them to be widely applied in molecular diagnosis, biological imaging, biosensing, targeted drug delivery, and regenerative medicine. With the development of DNA nanostructures in the past 40 years, tetrahedral DNA nanostructures (TDNs) stood out among various DNA nanomaterials, benefiting from their cellular membrane and tissue permeability, high yield, structural stability, negligible immunogenic response, and multifunctional editability.^13^

TDNs, also named tFNAs, were introduced by the Turberfield group in the early 2000s.^50^ Compared with other complicated DNA structures, tFNAs are some of the simplest and most specialized DNA polyhedrons. They can be easily synthesized with a yield of approximately 90%. Mao et al. reported that the assembly yield is lower when the size of the target structure is larger. For example, the yields of DNA dodecahedrons and buckyballs are 76% and 69%, respectively; in contrast, the yield of a DNA tetrahedron is approximately 90%.^45^ Besides their markedly high yields, three other prominent characteristics make tFNAs some of the most attractive, influential, and promising DNA nanomaterials in biomedical research. First, whether a material can enter the cell is the most crucial step for in vivo and in vitro applications. tFNAs can autonomously enter cells in large quantities in the absence of any functional molecules, a trait that makes them unique compared with other 2D or 3D DNA nanostructures. Considering the negative charges present on the tFNA surface, Fan and his colleagues used a single-particle tracking technique to study the cellular endocytosis of tFNAs. They suggested that tFNAs are assisted by caveolin to penetrate the cell membrane, and their lysosome internalization is facilitated by tubulin.^51^ Second, in addition to the non-toxic and negligible immunogenicity of tFNAs, it has shown positive effects on various types of mammalian cells such as RAW264.7, adipose stem cells (ASCs), chondrocytes, and L929 fibroblast-like cells at a relatively low concentration (250 nmol·L^−^^1^).^52–55^ Third, the precise programmability and controllable reversibility of tFNAs make them an ideal and promising carrier. tFNAs can be functionalized via simple modification by adding oligonucleotides or anti-oligonucleotides at the middle or end of the ssDNA,^56–59^ inserting functional small molecules into the duplex DNA through electrostatic adsorption,^60,61^ or chemical cross-linking between two ssDNA strands via complementary base pairing.^62–64^ tFNAs have been a research hotspot in the biomedical field, including bioimaging, biosensing, molecular diagnosis, gene delivery, disease treatments, and regenerative medicine, based on these unique characteristics. In this review, we will discuss the design, fabrication, and characterization of tFNAs. We will also touch on conventional tFNAs and the various processes regarding tFNA modification with different chemical moieties and biomolecules. Finally we will also discuss the applications of tFNAs in the regulation and treatment of various diseases, accompanied with a future perspective on applications of stimuli-responsive tFNAs.

## tFNAs

### Design and fabrication of tFNAs

tFNA, the simplest Platonic solid, has been successfully formed through a single-step synthesis firstly proposed by the Turberfield group.^50^ tFNAs possessing six edges and four vertexes were self-assembled from four specifically designed oligonucleotides (Table S1). Each oligonucleotide is composed of three different sequences, which complement the other three oligonucleotides based on Watson-Crick rules, suggesting that each strand of the tFNA was composed of DNA double helices. Importantly, to maintain the 60° angle of each corner between adjacent edges, unhybridized “hinges” need to be present. With their perfect programmability and high predictability, the four component ssDNAs were added at equimolar quantities in TM buffer (10 mM Tris, 20 mM MgCl_2_, pH 8.0), annealed (95 °C for 10 min, and quickly cooled to 4 °C for 20 min), and self-assembled into elegant tetrahedral cages (Fig. S1a). In addition, the yields of pure tFNAs without secondary structures can reach as high as 90%. Since the first tetrahedron with a length of 17 bp was introduced, five other types of tFNAs with different sizes (tFNA-7, tFNA-13, tFNA-21, tFNA-26, tFNA-37) were synthesized according to the Turberfield design principles (Fig. S1d).^65–67^ The edge of each tFNA with a different size contains a corresponding number of base pairs. Notably, tFNAs formed from four 63-mer oligonucleotides had edges with lengths containing 20 base pairs, separated by a single nucleotide at every vertex for sufficient flexibility, are commonly used by our research group. Compared with the other five types (tFNA-7, -13, -17, -26, and -37), tFNA-21 has a higher synthesis efficiency, fewer agglomerates, and is more stable in cell lysates or 10% fetal bovine serum for 12 h. Furthermore, they also show more positive effects on ASCs endocytosis (Fig. S1e), proliferation (Fig. S1f), and migration (Fig. S1g).^68^

Aside from single-strand annealing, two other uncommon methods are used for synthesizing tFNAs: 3-arm-junction hybridization (Fig. S1b)^26,69^ and scaffold folding (Fig. S1c).^70,71^ To fabricate a well-defined tFNA via 3-arm-junction hybridization, four identical copies of three-point-star motifs with two complementary single-stranded overhangs at the peripheral ends need to be gathered together to assemble a tetrahedron based on the complementary pairing of the sticky ends.^26^ Another approach is the top-down strategy reported by Bathe et al., a single-stranded scaffold designed according to the target structure where staple strands that filled the scaffold were hybridized into a robust tetrahedron.^70^ These two procedures for synthesizing tFNAs are not widely used because of their cost and yield. Hence, we will mainly discuss DNA tetrahedron assembly via single-strand annealing in this review.

### Cell membrane and tissue penetration

**Fig. 1:**
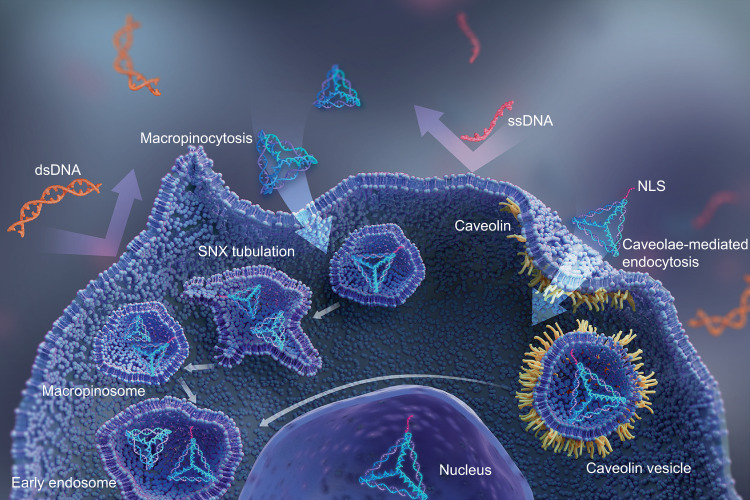
Cellular endocytosis of tFNAs. Naked DNA molecules (single- or double-stranded) could not autonomously permeate the cell membranes, while tFNAs could enter the cell; the tFNAs that functionalized with the nuclear localization sequences (NLSs) could enter the nucleus

Naked DNA molecules, which are genetic materials found in nature with highly negative charges, could not autonomously permeate the cell membranes with the same surface charges, either in the single- or double-stranded forms. In the beginning, researchers have used transfection agents to compensate for the charge of the nucleic acids or specifically targeted motifs that bind to the cell surface to facilitate the endocytosis of nucleic acids. For example, Mao and colleagues illustrated that DNA nanotubes functionalized with specific folate acid-targeting receptors in various cancer cells and Cy3 were effectively internalized by cancer cells for fluorescence imaging and cell sorting.^72^ Subsequently, with the rapid development of DNA nanotechnology, a series of DNA nanostructures with different dimensions have been fabricated by combining multi-stranded junction structures, using the scaffolded-stables technique, or through rolling-circle replication so they can penetrate the cell membrane. Surprisingly, Turberfield et al. have revealed that cultured mammalian cells could substantially take up tFNAs in the absence of any auxiliary ligands or agents, signifying that pure DNA nanostructures of certain geometries can autonomously pass through the cell membrane of live cells regardless of their surface charges (Fig. 1).^73^ Fan and his group also observed that pure tFNAs modified with unmethylated CpG motifs could noninvasively and efficiently enter immune cells.^56^

Numerous scientists have repeatedly confirmed the satisfactory cellular endocytosis of tFNAs, although the underlying mechanism of such internalization remains unclear. In 2014, an advanced single-particle tracking technique was employed to observe the endocytosis of tFNAs in live cells. Compared with other DNA nanostructures, tFNAs actively approached the cell membrane through their vertex by adjusting their orientation to reduce the electrostatic repulsion and let the uneven charge of the tFNA be redistributed across the membrane surface. After entering a cell via caveolin in the membrane, tFNAs were transported into the lysosome via microtubules in a highly ordered manner, maintaining structural stability in the cell cytoplasm for up to 12 h (Fig. 1).^51,74^ Furthermore, Shan et al. reported that tFNA attacked the membrane surface and rotated themselves, embedding one of their corners into the membrane to bind with receptors, completing the wrapping and internalization as a “corner attack.” They also emphasized that different sizes of tFNAs possess different rotation freedoms, accounting for the differences in the mechanisms of cell entry. Hence, the corner attack and orientation adjustment of tFNAs play a crucial role in the caveolin-mediated endocytosis pathway, consistent with the results of previous studies.^67,74^

It should be noted that there is another important protein besides caveolin-1 associated with the endocytosis of tFNAs, the micropinocytosis-related protein sorting nexin 5 (SNX5). SNX5 also mediates the internalization of tFNAs as observed by combining drug affinity responsive target stability (DARTS) with liquid chromatography/tandem mass spectrometry (LC-MS/MS). Li et al. reported that the cellular uptake of tFNAs was mediated via caveolae-dependent endocytosis and SNX5-associated micropinocytosis, which was confirmed through caveolin-1- and SNX5-knockout experiments, further corroborating previous findings.^75^ In addition, to change the fate of tFNAs in the lysosome for facilitating the delivery of various genes, drugs, and molecules, tFNAs were modified with nucleus-targeting signaling peptides through a “click” reaction, eventually being transported into the nucleus.^51^

**Fig. 2:**
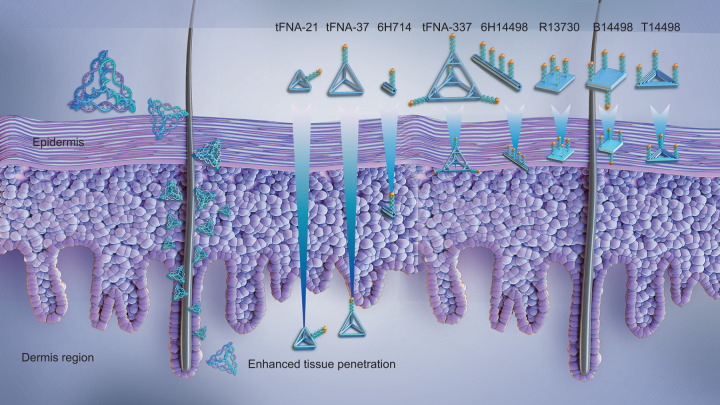
Enhanced tissue penetration of tFNAs. tFNA-21 reached ~350 µm beneath the skin surface; it showed the greatest penetration ability compared with the other seven types of nucleic acid frameworks with various spatial nanostructures

Apart from their cellular penetration ability, tFNAs also possess the capacity to penetrate whole tissues, which could expand their application as a drug delivery vehicle. The tissue penetration capacity of drug carriers is closely related to their physical parameters, such as size, morphology, charge, and material composition. Fan and his colleagues designed highly ordered framework nucleic acids (FNAs) with distinct shapes and sizes to verify their transdermal penetration ability via penetrating skin explants from mice and humans. Their results demonstrated that the penetration ability of these FNAs vehicles was greatly dependent on their sizes, as observed via skin histology. Briefly, 17-nm-long tetrahedral FNAs could reach the deepest region (~350 µm) from the skin periphery, showing the greatest penetration, while FNAs only reached ≤75 nm, effectively contacting the dermis. Remarkably, the FNAs could also maintain structural stability during skin penetration (Fig. 2). The penetration ability of tFNAs could be enhanced by functionalizing with doxorubicin (DOX), as it has been shown that tFNA-DOX systems could accumulate drugs 2-fold and effectively inhibit tumor growth.^66^ In addition to penetrating the skin, tFNAs modified with C-C chemokine receptor 2 (siCCR2) exhibited the ability to cross the blood-brain barrier (BBB) in a mouse model of intracranial hemorrhage (ICH).^76,77^ Therefore, the exceptional cellular membrane and tissue penetration of tFNAs make them an excellent and promising drug carrier for biomedical applications.

### Regulation of cell biological behaviors

#### Enhancement of cell proliferation, migration, and differentiation

An increasing amount of evidence has demonstrated that DNA nanostructures play a vital role in biomedical fields such as biosensing, bioimaging, molecular diagnosis, and drug delivery. Turberfield et al. first created tFNAs in 2004 and notably reported their internalization into mammalian cells in 2011.^50,73^ Although Fan and his groups had in-depth studies of tFNAs in biological sensing and imaging,^65,78,79^ the interaction between cells and tFNAs has almost been unstudied for a long time. According to the natural ability of DNA to autonomously enter mammalian cells without the aid of adjuvant agents, in 2016, our group attempted to consider the biological effects of pure tFNAs after they are endocytosed at different concentrations ranging from 62.5 nmol·L^−1^ to 500 nmol·L^−1^ .^53^ We reported in our first study that cell proliferation was markedly enhanced in the presence of tFNAs (250 nmol·L^−1^) by regulating the Wnt/β-catenin signaling pathway. Real-time cell analysis (RTCA) and cell counting kit (CCK8) assays were applied to evaluate cell proliferation, revealing that tFNAs promoted the proliferation of mouse L929 fibroblasts in a concentration-dependent manner (<500 nmol·L^−1^). Cyclin-dependent kinase-like 1 (CDKL1), which mediates cell entry into the S phase of the cell cycle, was upregulated upon exposure to tFNA, as revealed in the microarray analysis.

Furthermore, tFNA has positive effects on enhancing the proliferation of various cell types, such as chondrocytes,^80^ various stem cells (neural stem cells [NSCs],^81^ ASCs,^52^ mesenchymal stem cells [MSCs],^63^ and human periodontal ligament stem cells [PDLSCs]^82^), and myoblasts,^83^ among others. Lin and his colleagues also explained the mechanism of tFNA-enhanced cell proliferation from the perspective of epigenetics. The results of an epigenetics microarray revealed a few differentially methylated regions regulated the expressions of different genes upon exposure to tFNAs at a concentration of 250 nmol·L^−1^. Importantly, tFNA treatment induced the hypermethylation of the Dlg3 gene promoter, enhanced cell proliferation, and inhibited cell apoptosis.^52^

In 2016, our group reported that cell migration, aside from cell proliferation, was also induced by tFNA treatment, as we first detected in ASCs.^54^ After the ASCs internalized the tFNAs, the long noncoding RNA (lncRNA) XLOC 101623 was downregulated, activating the mRNA expression of *Tiam1* and *Rac1*, subsequently activating the RHOA/ROCK2 signaling pathways to promote cell migration. It is well-known that cell migration plays a crucial role in wound healing and tissue regeneration. Hence, keratinocytes (HaCaT cell line) and fibroblasts (HSF cell line), which are cells involved in the complex cutaneous wound healing process, were co-cultured with tFNAs. It was found that tFNAs promoted the proliferation and migration of these cells. Moreover, in vivo experiments demonstrated that tFNAs could accelerate the healing of cutaneous wounds and reduce the presence of scars.^84^ Significant promotion of cell migration was also found in endothelial cells (ECs),^85,86^ human corneal epithelial cells,^87^ chondrocytes,^60,88^ NSCs,^85^ synovium-derived MSCs (SMSCs),^89^ and Schwann cells (SCs)^90^ in the presence of tFNAs.

The differentiation of stem cells plays a vital role in tissue engineering. Numerous studies by the Lin and Guo research groups have reported that tFNAs can regulate the differentiation of stem cells. In the nervous system, tFNAs showed neuroprotection and neuroregeneration ability. After the primary NSCs were treated with tFNAs, their proliferation was enhanced. Moreover, tFNAs could also promote transplanted NSCs to differentiate into neurons and oligodendrocytes and inhibit them from differentiating into astrocytes, facilitating the neural tissue formation in the injured spinal cord.^91^ In the immune system, tFNAs were shown to have excellent immunomodulatory capacities, which can regulate the differentiation of B cells and T cells by influencing cytokine secretion and signal transducer and activator of transcription (STAT) signaling.^90,92^ In the cartilage, Guo and his colleagues have highlighted that tFNAs showed potential as DNA nanostructures that could promote cartilage tissue engineering, as they could increase the ability of SMSCs to proliferate, migrate, and differentiate into chondrocytes.^89,93^ In the skeletal system, pure tFNAs could dramatically enhance the osteogenic differentiation of ASCs by regulating the Wnt/β-catenin signaling pathway,^94^ the osteo/odontogenic differentiation of dental pulp stem cells (DPSCs) accompanied by activating the Notch signaling pathway,^95^ and the osteogenic capacity of PDLSCs.^82^ In addition, tFNAs functionalized with miR-2861 targeted the histone deacetylase 5 (HDAC5) in MSCs, facilitated the osteogenic differentiation of MSCs, and inhibited the expression of HDAC5, eventually promoting bone repair.^63^

#### Inhibition of cell apoptosis

Aside from the above-mentioned properties of regulating cell proliferation, migration, and differentiation, there is another characteristic of tFNAs: anti-apoptosis. Apoptosis, also known as programmed cell death, is a cellular process involving a series of significant changes in morphology and metabolic activity to ultimately induce cell death, playing a critical role in the development and normal function of an organism.^96^ The main characteristics of cell apoptosis include nuclear shrinkage, an abnormal cell cycle, and the expression of apoptosis mediators. Our research group has found that tFNAs could effectively suppress the cell apoptosis induced by excessive reactive oxygen species (ROS) production and inflammatory responses.^52,60,93,97,98^ Excessive ROS production in organisms is mainly attributed to uncontrolled oxidative stress. For oxidative stress-induced apoptosis, Cai et al. employed tFNAs to reduce the production of ROS in retinal ganglion cells (RGCs) injured using tert-butyl peroxide (TBHP), regulate the expression of oxidation-related enzymes to protect RGCs from oxidative stress, and affect the expression of apoptosis-related proteins, all of which could inhibit the apoptosis of RGCs.^98^ In the myocardial ischemia-reperfusion injury (MIRI) model, tFNA was vital in easing oxidative damage and mediating the expression of apoptosis-related genes (including *BCL2*, *BAX*, and *Caspase-3*) to inhibit cell apoptosis induced by reperfusion.^97^ Unsurprisingly, the expression of the antioxidative enzyme heme oxygenase-1 (HO-1) was upregulated by tFNAs to attenuate ROS generation and oxidative stress, inhibiting the apoptosis of RAW264.7 cells stimulated with lipopolysaccharide (LPS).^52^

Similarly, the Nrf2/HO-1 signaling pathway regulates oxidative stress in osteoarthritis (OA). Some studies reported that tFNA treatment in an IL-1β-induced OA model suppressed chondrocyte apoptosis by attenuating oxidative stress and affecting the BCL2/BAX/caspase-3 pathway.^60,99^ In addition, Wu and his group suggested that poly(ADP-ribose) polymerase (PARP) was involved in cell apoptosis triggered by cisplatin in an acute kidney injury (AKI) model. tFNAs have been shown to suppress the cleavage of PARP, reduce cell apoptosis, and downregulate glutathione peroxidase 4 (GPX4) expression and ROS production to prevent ferroptosis caused by RSL3.^100^ For inflammation-induced apoptosis, the primary goal is to inhibit the expression of inflammatory-related factors such as interleukin-1β (IL-1β), IL-6, and tumor necrosis factor α (TNF-α). A recent report revealed that neuronal (SHSY-5Y cells) apoptosis induced by ischemic stroke was inhibited in the presence of tFNAs by suppressing inflammation.^93^ Severe acute pancreatitis (SAP), an inflammatory disease of the pancreas characterized by a systemic inflammatory response, can cause tissue injury and necrosis. The effective inhibition of inflammation and suppression of pathological cell death needs to be done to reverse or prevent the progression of SAP. tFNAs can efficiently decrease the expression of inflammatory cytokines, affect the expression of apoptosis-associated proteins to alleviate cell apoptosis, and prevent SAP progression and multiorgan injury.^101^ Mitochondrial-mediated apoptosis is another critical way to trigger cell apoptosis aside from oxidative stress- or inflammation-induced apoptosis. tFNA treatment regulated the expression of Bcl-2, Bax, and Caspase-3, which are associated with mitochondrial apoptosis, to inhibit apoptosis and cure various diseases.

For example, in Alzheimer’s disease (AD), tFNAs notably attenuated PC12 cell apoptosis at a concentration of 250 nmol·L^−1^ by regulating the expression of Bcl-2, Bax, and Caspase-3.^102,103^ In Parkinson’s disease (PD), tFNAs showed extraordinary neuroprotective and neurorestorative effects by inhibiting the apoptosis of PC12 cells through regulation of the expression of the three genes previously mentioned. Similar therapeutic effects were observed in subarachnoid hemorrhage (SAH),^104^ type 2 diabetes mellitus (T2DM),^100^ and steroid-associated osteonecrosis (SAON).^62^ Importantly, DNA hypermethylation of the *Dlg3* gene promoter was also related to suppressing ASC apoptosis upon exposure to tFNAs.^52^ These merits of tFNAs further facilitate their wide applications in the biomedical field (Table 1).

**Table 1:** Application of tFNAs for regulating cellular and biological behaviors

Regulations of cell behaviors	Signal pathway	Relevant gene/protein expression	Cell type	References
**Enhance** proliferation	Wnt/β-catenin	β-catenin ↑ , Lef-1 ↑ , cyclin D ↑	Mouse L929 fibroblasts	^53^
**Promote** osteogenic potential and proliferation	Wnt/β-catenin	ALP ↑ , Runx2 ↑ , OPN ↑ , β-catenin ↑ , Lef-1 ↑ , cyclin-D ↑	Adipose stem cells (ASCs)	^94^
**Promote** proliferation **maintain** phenotype	Wnt/β-Catenin, Notch	β-catenin ↑ , Lef-1 ↑ , cyclin-D ↑ , COL-II ↑ , aggrecan ↑ , Notch1/3 ↓ , Hes1↓	Chondrocytes	^80^
**Promote** proliferation and differentiation	Wnt/β-catenin, Notch	β-III-tubulin ↑ , β-catenin ↑ , Lef-1 ↑ , cyclin-D ↑ , Notch1 ↓ , Hes1 ↓ , Hes5↓	Neuroectodermal (NE-4C) stem cells	^81^
**Enhance** proliferation, **reduce** apoptosis	N/A	Dlg3 ↓ , caspase3 ↓ , Bax ↓ , Bcl-2↑	ASCs	^52^
**Promote** proliferation and migration	P38, ERK1/2	p-P38 ↑ , p-ERK1/2↑	Human corneal epithelial cells (HCECs)	^87^
**Promote** proliferation, migration	N/A	N/A	Keratinocytes (HaCaT cell line), fibroblasts (HSF cell line)	^84^
**Enhance** proliferation, migration and chondrogenic differentiation	Wnt/β-catenin, TGF/Smad2/3	β-catenin ↑ , Lef-1 ↑ , cyclin D1 ↑ , COL-II ↑	Synovium-derived mesenchymal stem cells (SMSCs)	^89^
**Enhance** proliferation, migration, secretion of functional proteins	NGF/PI3K/AKT	Nerve growth factor (NGF) ↑ , p-Pi3k ↑ , p-Akt↑	Schwann cells	^90^
**Enhance** proliferation and osteogenic differentiation	Wnt/β-catenin	Runx2 ↑ , OPN ↑ , Lef-1 ↑ , β-catenin ↑ , GSK-3β ↓	Periodontal ligament stem cells (PLSCs)	^159^
**Promote** migration	TIAM1/RAC1, RHOA/ROCK2	lncRNA XLOC 101623 ↓ , Tiam1 ↑ , Rac1 ↑ , RhoA ↑ , Rock2↑	ASCs	^54^
**Modulate** motility	RHOA/ROCK2	RhoA ↑ , Rock2 ↑ , Vinculin↑	Chondrocytes	^88^
**Enhance** migration	RHOA/ROCK2	RhoA ↑ , Rock2 ↑ , Vinculin↑	NE-4C stem cells	^170^
**Enhance** osteogenic/odontogenic differentiation	Notch	Runx2 ↑ , OPN ↑ , Notch1 ↑ , Hes1 ↑ , Hey1↑	Dental pulp stem cells (DPSCs)	^95^
**Promote** differentiating into neurons and oligodendrocytes	N/A	MBP ↑ , GFAP ↓	Neural stem cells (NSCs)	^91^
**Regulate** differentiation of B and T cells	N/A	TGF-β ↑ , IL-10↑	T/B cells	^92^
**Decrease** pro-inflammatory cytokines and cellular ROS, **promote** osteogenic differentiation	MAPK/ERK	TNF-α ↓ , IL-6 ↓ , IL-1β ↓ , ERK ↓ , JNK ↓ , P38 ↓	LPS-induced PLSCs	^82^
**Alleviate** apoptosis	TLR2-MyD88-NF-κB	ROS ↓ , Erythropoietin↑	SHSY-5Y cells	^93^
**Prevent** oxidative damage and apoptosis	Akt/Nrf2	ROS ↓ , Bax ↓ , caspase-3 ↓ , pAkt/Akt ↑ , Nrf2 ↑ , HO-1↑	H9c2 cells	^97^
**Alleviate** oxidative stress-induced apoptosis	AKT/Nrf2	ROS ↓ , p-Akt/Akt ↑ , Nrf2 ↑ , HO-1↑	Retinal ganglion cells (RGCs)	^98^
**Reduce** apoptosis, **inhibit** oxidative stress, **enhance** autophagy	BCL2/BAX/caspase-3, Nrf2/HO-1	Bax ↓ , caspase-3 ↓ , Bcl-2 ↑ , Nrf2 ↑ , HO-1↑	Chondrocytes	^99^
**Preserve** from apoptosis	BCL2/BAX/caspase-3	Bax ↓ , caspase-3 ↓ . Bcl-2↑	Pancreatic cells	^101^
**Protect and rescue** Aβ25 35-induced apoptosis	ERK1/2	p-ERK1/2↑	PC12 cells	^102^
**Decrease** hemin-induced apoptosis	BAX/BCL-2	Bax ↓ , caspase-3 ↓ , Bcl-2↑	Hemin-induced brain microvascular endothelial cells (BMECs)	^104^
**Ameliorate** apoptosis	N/A	ROS ↓	HepG2 cells	^100^

### tFNA functionalization via multiple programmabilities

Considering all the excellent characteristics of tFNAs, researchers have recently focused on multifunctional tFNAs modified in several ways, such as sequence extending, small molecule intercalation, drug encapsulation, and cohesive end complementary pairing. Hence, various therapeutic drugs (nucleic acid molecules, anti-cancer drugs, traditional Chinese medicine monomers, and functional proteins) or biosensing molecules (fluorescent dyes, bioligand molecules) could be carried into mammalian cells and organisms by tFNAs. The processes and applications of the four main modifications are summarized below (Table 2).

**Table 2:** Modifications of tFNAs and their applications for the delivery of therapeutic molecules

Modification type	Functional molecules	Classification	Biomedical application	Diseases	References
Sequences extension	Toll-like receptor 9 (TLR9)	Receptor	Immunostimulation	N/A	^56^
	AS1411	Aptamer	Anti/Target-cancer	Breast cancer, melanoma, cervical cancer, lung cancer	^58,59,106–109^
	Anti-HER2 aptamer (HApt)	Aptamer	Anti/Target-cancer	HER2-positive breast cancer	^110^
	GMT8, Gint4.T	Aptamer	Target-cancer	Astroblastoma	^112^
	Freestanding probe left by long ssDNA	DNA	Ultrasensitive detection of miR-155	N/A	^116^
	Antisense oligonucleotide against miR132 (miR132-ASO)	Antisense oligonucleotide	Promotes the differentiation of dopaminergic neurons	Neurodegenerative disease	^113^
	Antisense oligonucleotide against c-Met mRNA (c-Met mRNA-ASO)	Antisense oligonucleotide	Anti-cancer	N/A	^114^
	DNAzyme-13 (Dz13)	Oligonucleotide	Cleave targeted mRNA (c-Jun)	Epidermoid carcinoma	^64^
	miR-355-5p	miRNA	Bone regeneration	Steroid-associated osteonecrosis (SAON)	^62^
Sticky-end hybridization	Au@Cu_2-x_S@polydopamine nanoparticle (ACSP)	All-in-one nanoagent	Cancer theragnosis, treatment	Cervical cancer	^115^
	N-(ε-malemidocaproyloxy) succinimide ester (EMCS)	Ligand	Target-cancer	HER2-positive breast cancer	^117^
	Cu-NMOF@PtNPs/HRP	All-in-one nanoagent	Ultrasensitive detection of miR-155	N/A	^118^
	H1&H2	Hairpin-structured DNA	Sensitive imaging of intracellular pH and targeted mRNA	N/A	^119^
	siCTR1	siRNA	CTR1 mRNA targeting	Pancreatic cancer	^122^
	C3, C6	siRNA	CSFV genome targeting	Classical swine fever	^123^
	siApoB1	siRNA	ApoB1 mRNA Targeting	Hypercholesterolemia	^124^
	siRNAs	siRNA	Tumor relevant gene silence	Cervical cancer	^125^
	*ftsZ*-targeted antisense peptide nucleic acid (*ftsZ*-asPNA)	Peptide nucleic acid	Anti-bacterial	MRSA-caused infections	^126^
Intercalation	Doxorubicin (DOX)	Chemotherapeutic drug	Anti-cancer	Breast cancer, PTK7-positive tumor	^127–131^
	56MESS	Chemotherapeutic drug	Anti-cancer	Squamous-cell carcinoma	^133^
	Curcumin	Chinese medicine monomer	Anti-inflammation	Gouty arthritis	^135^
	Resveratrol (RSV)	Chinese medicine monomer	Anti-inflammation	Insulin resistance	^136^
	RuPOP	Metal complex	Anti-cancer	Liver cancer, cervical cancer, breast cancer, melanoma	^137^
	Dye	Fluorescent molecule	Detection and imaging	N/A	^138^
	SYBR Green I	Fluorescent molecule	Detection and imaging of IgG	N/A	^139^
Encapsulated	Cytochrome *c*	Cytochrome	Induce apoptotic protease cascade	N/A	^140^
	RNase A	Chemotherapeutic drug	Anti-cancer	N/A	^141^
	Melittin	Peptide	Anti-cancer	N/A	^142^

#### Extended sequence type

As previously reported,^94,105^ tFNAs could self-assemble from four specific DNA oligonucleotide sequences. According to homopolymeric oligonucleotide end ligation via terminal transferase, different oligonucleotides (DNA, RNA, and other nucleotides) can be linked at the 5′- or 3′-ends of ssDNA. Then, four normal ssDNAs and functional ssDNAs can form the multifunctional tFNA with targeted and therapeutic efficacy via the same PCR procedure (Fig. S2a). Li and his colleagues (2011) first linked the CpG motif, a highly immunostimulatory therapeutic oligonucleotide, into tFNAs to bind with Toll-like receptor 9 (TLR9) and enhance its immunostimulatory effects.^56^ It is important to note that several nucleotides (A or T) should be added to connect the functional groups and ssDNA without interrupting the formation of the 3D spatial structure of tFNAs, thereby confirming the safety of the functional domain. Since there are four ssDNAs used to synthesize tFNAs, there are four loading sites to choose from during the design of the molecule. AS1411, an aptamer conjugated with nucleolin overexpressed on the surface of cancer cells, was attached to tFNA at one vertex, and was reported to improve the efficiency of cellular endocytosis to deliver therapeutic and imaged tFNA.^58,59,106–109^ Similarly, Ma et al. adopted an anti-HER2 aptamer carried by tFNAs to target HER2-positive breast cancer cells. These HER2-tFNA complexes mediated the breast carcinoma cell apoptosis and inhibited their growth.^110,111^ The GMT8 and Gint4.T aptamers that specifically bind to U87MG-loaded tFNAs modified with paclitaxel enhanced apoptosis and suppressed the proliferation, migration, and invasion of U87MG cells.^112^ Apart from CpG and targeting aptamers, therapeutic and biosensing RNAs (microRNAs,^62,112^ antisense oligonucleotides (ASOs),^113,114^ and DNAzymes^64^) can also be anchored on the ssDNA assembly to create functional tFNAs. When carried by tFNAs, the stability and cell and tissue permeability of these RNAs were enhanced even in complicated circumstances, overcoming biological barriers and improving their therapeutic efficacy. For example, microRNA335-5p (miR335-5p), which targets and inhibits *DKK1* expression to regulate the osteogenic differentiation of BMSCs and enhance new bone formation in rats, was linked to a ssDNA during the synthesis of tFNAs, showing an outstanding curative effect on SAON.^62^

#### Sticky-end hybridization type

Compared with the directly functional oligonucleotide extended type, anchoring nucleic acids with various functions on tFNAs via sticky-end hybridization may be a more practical approach (Fig. S2b). Functional molecules or groups could hybridize with ssDNAs or ssDNA extensions at the end or middle of their sequences. First, the 5′ or 3′ ends of ssDNAs are extended with a complementary oligonucleotide containing functional motifs. This approach has been demonstrated in biological sensing and disease diagnosis.^115–120^ miRNAs are an ideal potential biomarker for the diagnosis and prognosis of cancer. Hui et al. modified the vertex of tFNAs with a specific oligonucleotide that can hybridize with miR-155 overexpressed in breast cancer cells. This freestanding probe at the vertex of tFNAs can capture the target miR-155.^116,121^ Second, complementary hangs at the edges (one to six nucleotides) of tFNAs (middle or other non-vertex regions) could also hybridize with the overhangs of functional ssDNA strands.^59,122–124^ Lee and his colleagues achieved targeted tFNA-mediated siRNA delivery in vivo. Six siRNAs linked at the edges of tFNAs could improve their serum stability and reduce potential immune stimulation, facilitating their therapeutic efficacy via gene silencing in cancers.^125^ Third, overhangs bound to the vertices or edges of tFNAs have relatively poor stability compared with those incorporated into tFNAs. Antisense peptide nucleic acids (asPNAs) complementary to a region of one of ssDNAs and the other three ssDNAs facilitated the self-assembly of functional tFNAs (P-tFNAs) without changing their size, structure, and vector properties. P-tFNAs could enter methicillin-resistant *Staphylococcus aureus* cells (MRSA) without the aid of auxiliary molecules and inhibit the expression of *ftsZ* in a concentration-dependent manner.^126^ The key point of this approach is that the length of the functional molecules should be as short as a third of the ssDNAs that form the tFNAs. Thus, this delivery method is applicable to only a few functional DNA or RNA sequences.

#### Intercalation type

Intercalation modification is when functional molecules are embedded into the dsDNA helix of tFNAs via conjugation (Fig. S2c). Several functional groups (such as anti-cancer drugs,^85,127–134^ conventional Chinese medicine monomers,^60,135,136^ metal complexes,^137^ or fluorescent molecules^138,139^) could be intercalated into tFNAs for cellular delivery. DOX, a broad-spectrum anti-cancer drug, can interfere with macromolecular biosynthesis by embedding into the helix of DNA double strands.^131^ To mitigate the disadvantages of using DOX, such as its side effects, poor selectivity, multidrug resistance, and response release, tFNAs could be used to load DOX via chemical conjugation. DOX@tFNA complexes have been shown to effectively enter cells via endocytosis, overcome multidrug resistance, and induce cancer cell apoptosis.^109,128^ In addition to DOX, other anti-cancer drugs (PTX,^85^ platinum,^133^ and camptothecin^134^) can also be encapsulated using tFNAs via intercalation within the DNA double-stranded helix. Traditional Chinese medicine monomers are not widely applied in clinics due to their poor aqueous solubility, low stability, and inadequate cellular and tissue penetration. Delivery systems such as DNA nanomaterials are needed to enhance their stability and transport into cells, improve their therapeutic efficacy, and increase their bioavailability. Our research group built a tFNA-based Chinese medicine monomer delivery system loaded with functional monomers (resveratrol,^136^ wogonin,^60^ and curcumin^61^) via conjugation. Furthermore, metal complexes (such as ruthenium polypyridyl complexes [RuPOP]) and fluorescent molecules (SYBR Green I^139^ and other dyes^138^) have been intercalated into the dsDNA helix of tFNAs for use in cancer treatment and fluorescence detection and imaging, respectively.

#### Encapsulated type

Functionalization of the encapsulated type means that functional molecules were wrapped inside the caged structure of the tFNA (Fig. S2d). It was first reported by the Turberfield research group, which demonstrated that cytochrome C could be put inside the robust tFNA nanocage by binding it to a particular modification site on the ssDNA to stabilize its position. The central cavity of tFNA was calculated to have a radius of approximately 3 nm, accommodating a small spherical molecule (for example, a < 60 kDa globular protein). An apoptotic protease cascade was induced by a functional tFNA encapsulating cytochrome C (molecular weight: 12.4 kDa).^140^ Following this novel approach, Xiang and colleagues accommodated the native therapeutic protein (RNase A: ~13.4 kDa) inside the inner cavity of a tFNA via a reversible chemical bond. It has been shown that the ligase-assisted sealing of tFNA ends makes these nanostructures highly stable against nuclease digestion. As an anti-cancer therapeutic protein, RNase A can suppress protein synthesis and induce cancer cell apoptosis by cleaving intracellular RNA. Thus, tFNA-RNase A complexes can enter cells via endocytosis and successfully release RNase A, degrading cellular RNA to induce cancer cell apoptosis.^141^ Furthermore, a dynamic and active targeting tFNA was designed and developed by Tian et al. to deliver melittin.^142^ The tFNA-melittin complex, also named nanobee, could selectively release melittin from the tFNA structure once it underwent a conformational change stimulated by its target proteins on the cell membrane. The nanobee has been shown to have a stronger selectivity and higher cytotoxicity against cancer cells than free melittin molecules.

### Enhancing the stability of tFNAs

**Fig. 3:**
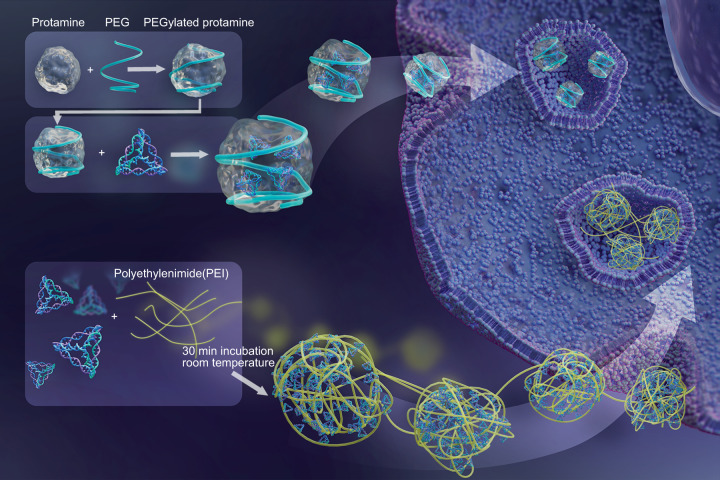
Enhanced stability of tFNAs. Polymer ethyleneimine (PEI) and PEGylated protamine were used to enhance the stability and internalization rate of tFNAs

The natural biocompatibility, structural stability, editable functionality, and cellular and tissue permeability of tFNAs make them ideal and promising for broad disease diagnosis and drug delivery applications. However, several problems still need to be solved so they can be used in in vivo applications, such as the need for increased resistance against numerous nucleases, enhancement of cellular endocytosis, and extension of their circulatory time. Our research group introduced two cationic polymers, which are classic carriers for gene delivery, to tFNAs: polyethyleneimine (PEI)^143^ and PEGylated protamine (Fig. 3).^144^ We explored if these polymers could further enhance the stability of tFNAs in complicated physiological processes or pathological conditions, during cell and tissue membrane penetration, and in lysosomal escape. PEI (25 kDa, branched) and tFNA were combined with PEI/TDN complexes, relying on electrostatic forces via a facile one-pot synthesis approach. The modification of tFNAs with PEI enhanced their systemic stability, endocytosis efficacy, and lysosome-escape ability.^143^ However, PEI was reported to be cytotoxic as it inhibits cell viability,^145^ limiting its applications. Protamine and other cationic peptides for gene transfection modified using poly(ethylene glycol) (PEG) can absorb negatively charged tFNAs via electrostatic adsorption, forming PEG-protamine-tFNA complexes.^144^ With the aid of PEGylated protamine, tFNAs exhibited a more significant positive influence on cellular endocytosis, cell proliferation, and lysosome escape in three tissue-derived cells. The charge neutralization by cationic polymers can reduce the nonspecific clearance and increase the circulation time of tFNAs, enabling further in vivo applications.

### In vivo distribution

As a potential delivery vehicle for functional molecules and therapeutic drugs, exploring the in vivo distribution of tFNAs is the prerequisite for its in vivo applications. There are almost no systematic studies on the in vivo distribution of tFNAs. Only a few studies have reported that tFNAs with robust 3D controllable nanostructures (~10 nm) are preferred. Our research team, using an in vivo imaging system (IVIS), revealed that the blood circulation time of tFNAs in mice was just ~1 h after intravenous injection. Fluorescence signals from fluorophores (tFNA-Cy5 constructs) were enhanced in the bladder over time.^55,91^ Similar results were suggested by Tian et al., as they reported that tFNAs labeled with fluorescent moieties could be quickly absorbed and accumulated by the renal, and are subsequently cleared by the kidney.^146^ Combined with the various merits and preferential renal accumulation of tFNAs, Zhang and her colleagues explored their therapeutic efficacy in an animal model of rhabdomyolysis acute kidney injury (RM-AKI). They showed that tFNAs exhibited a better therapeutic ability on injured kidneys via ROS scavenging and the inhibition of cellular apoptosis.^147^ Apart from the kidney, other crucial organs such as the liver and gall bladder were also observed to accumulate fluorescence signals after the tail vein injection of tFNAs-Cy5. However, the fluorescence intensity of these two organs was lower than that in the kidney. In addition to intravenous or intraperitoneal injection, in situ injections of tFNAs were also applied in brain,^76^ keen joint,^60^ and ankle joint.^61^ In situ injections can realize the effective concentration of tFNAs and impart a more prolonged therapeutic time. Compared to passive distribution, the targeted delivery of tFNAs and functional molecules can be achieved through various chemical modifications. AS1411 aptamers, which specifically bind with nucleolin expressed on the membranes of most carcinoma cells, can be used to modify tFNAs and target and accumulate in tumor tissues.^58,59^ Besides AS1411 aptamers, folic acid and tumor-penetrating peptides can also be loaded by tFNAs to target tumors. Due to the versatility of tFNAs in terms of modification, specific target requirements can be met.

## Application of tFNAs in regenerative medicine

In recent biomedical engineering, regenerative medicine dominates in these fields, attracting the most attention from many researchers. Regenerative medicine, which replaces or regenerates diseased or injured tissues or organs by delivering cells and tissue constructs and activates innate healing responses by therapeutical molecules to acquire normal structure and function, is one of the fastest-evolving interdisciplinary disciplines to address various medical challenges.^148^ Ultimately, this approach aims to restore the function of cells that are damaged, aging, and lost by inducing self-healing or replacing them with new ones. There are three major elements involved in regenerative medicine: cells, scaffolding, and bioactive signaling molecule.^149^ Although signaling molecules are responsible for stimulating the regenerative process and regulating cellular processes and scaffolding provides a 3D extracellular matrix to induce tissue formation, cells are the most significant for regenerative medicine compared with signaling molecules and scaffolding.^150^ Obviously, it is quite important to enhance cell activity to develop regenerative medicine. For example, the cell ability to migrate, proliferate, and differentiate in the damaged or injured tissues is terribly low. Therefore, enhancing the natural healing potential of cells by using therapeutic molecules could achieve tissue regeneration. Biomaterials, particularly DNA nanomaterials, maybe the best choice for enhancing cell activity. With the rapid development of 3D DNA nanostructures, tFNA could be used to treat and regenerate related tissue defects, especially in craniomaxillofacial tissue.

### Bone tissue regeneration

In the dental and craniofacial regions, bone tissue consists of four parts: the mandible, auditory ossicles, neurocranium, and splanchnocranium. The brain is protected by the neurocranium, and the face is supported by the splanchnocranium.^151^ Bone tissue regeneration plays an important role in treating bone defects caused by bone infection, genetic defects, accidental trauma, bone tumors, and other diseases. Craniofacial bone defects generally lead to deformities in varying degrees as well as motor and non-motor dysfunction (including speech, vision, hearing, brain function, chewing, and swallowing). It is universally known that bone regeneration is a highly complex and dynamic process involving cell proliferation, differentiation, migration, and matrix formation, accompanied by bone remodeling.^152^ Over the past few decades, bone substitutes (tissue-engineered, autogenous, heterogeneous, allograft, and artificial bone) implanted into bone defects comprised the conventional therapeutic approaches for patients who need these interventions.^153,154^ However, an insufficient donor supply of autogenous bone, postoperative infection, and the immune rejection of other bone substitutes severely decrease the cure rates of this disease. At present, a combination of biological materials such as DNA nanomaterials and stem cells has been widely applied to treat bone defects. With the swift development of DNA nanotechnology, advances have been made to apply DNA nanostructures, particularly tFNAs, to bone regeneration.

Stem cells are crucial for repairing and regenerating bone tissue. MSCs, ideal candidate pluripotent stem cells derived from the mesoderm, retain their self-renewal capability and differentiate into osteoblasts, chondrocytes, adipocytes, and other cell types when induced under specific conditions.^155,156^ The low survival rate and differentiation efficiency of MSCs in defective parts limit their applications. Hence, tFNAs possessing good biocompatibility were reported to positively affect cellular behaviors, including proliferation, migration, differentiation, and maintain the phenotype of cells.^157^ Li et al. demonstrated that tFNAs modified with miR-2861 enhanced the osteogenic differentiation of MSCs by promoting the bone-specific protein expression of HDAC5, facilitating bone repair and regeneration in a bone defect model.^63^ Similarly, miR335@tFNAs were reported to promote the proliferation and inhibition of MSC apoptosis, increase the expression of alkaline phosphatase, reduce the expression of lipid droplets, and improve the secretion of VEGF and the formation of vascular-like structures. In a SAON model, the bone defects induced by osteonecrosis were restored by new bone and neovascularity formation, synergistically reducing the prevalence of empty lacunae by regulating the Wnt signaling pathway after treatment with miR335@tFNAs/Li-hep-gel.^62^ In addition, another type of mesenchymal stem cell, ASCs, is also an ideal cell for bone regeneration applications. Shao and her colleagues found that tFNAs could remarkably improve the proliferation and osteogenic differentiation of ASCs by upregulating the expression of genes and proteins associated with osteogenic differentiation (ALP, Runx2, and OPN) and the Wnt signaling pathway (β-catenin, Lef-1, and cyclin-D). tFNAs best enhanced these parameters at a concentration of 250 nmol·L^−1^ .^94^

DPSCs with common mesenchymal stem cell characteristics have been indicated in alveolar bone tissue regeneration to form the dentin-pulp complex induced from HA/TCP scaffolds.^158^ Thus, DPSCs are especially suitable for bone and dental tissue regeneration. Zhou et al. demonstrated that the proliferation of DPSCs can be dramatically upregulated by activating the cell cycle upon exposure to tFNAs. The mRNA and protein expressions of ALP, RUNX2, and OPN were enhanced in the presence of tFNAs at a concentration of 250 nmol·L^−1^, showing that tFNAs promoted osteogenic differentiation in DPSCs. Notably, tFNAs can enhance the odontogenic differentiation of DPSCs, accompanied by an upregulation of DSPP expression, a key marker of odontogenesis. The Notch signaling pathway has also been shown to be tightly involved in the proliferation and osteo/odontogenesis of DPSCs, and are regulated by tFNAs.^95^

Furthermore, DPLSCs separated from the periodontal ligament have been confirmed to differentiate into osteoblast-like and cementum-like cells. Notably, periodontium-like connective, osteoid, and cementoid tissue can be naturally generated from cementum-like cells, suggesting that DPLSCs are crucial for reconstructing alveolar bone defects. tFNAs also showed promotive effects on the proliferation and osteogenic differentiation of DPLSCs by activating the Wnt/β-catenin signaling pathway; in contrast, β-catenin, Lef-1, and cyclin-D are key regulators of this pathway.^159^ These data suggest that tFNAs have potential applications for DPSC- and DPLSC-based bone and dental tissue regeneration.

**Fig. 4:**
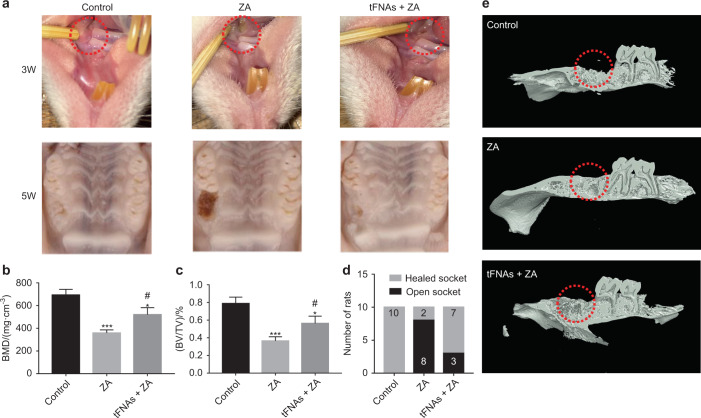
tFNAs enhance bone tissue regeneration. **a** The areas of the BRONJ-affected regions were smaller at 3 and 5 weeks after treatment with the tFNAs. **b** Bone remodeling showed that the tFNAs enhanced bone regeneration. **c**, **d** BMD and BV/TV statistical analysis of the samples from the three groups. **e** The healing scores of the samples from the tFNA-treated groups were higher than those of the samples from the control groups. Reproduced with permission.^161^ Copyright 2020, Royal Society of Chemistry

Bisphosphonate-related osteonecrosis of the jaw (BRONJ) is a serious maxillofacial complication caused by the exposure of bones in the mandible or maxilla to bisphosphonates (BPs) for more than eight weeks without radiotherapy. BRONJ is characterized by bone infection, necrosis, pain, and halitosis, terribly affecting the quality of life of patients with the disease.^160^ The main factor leading to BRONJ is oral operative processes, including tooth extraction, periapical procedures, and implant placement. However, there is currently no available treatment for this disease. Considering the anti-inflammatory, anti-oxidation, and angiogenetic enhancement abilities of tFNAs, researchers have eagerly explored whether tFNAs could treat BRONJ. tFNAs exhibited remarkably effective therapeutic efficacy on this disease through three aspects (Fig. 4).^161,162^ First, the zoledronic acid (ZA)-induced inhibitory effects on osteoclast (OC) differentiation and maturation could be effectively reversed. In turn, this regulates the expression of C-fos and NFATc1, which are markers of OC differentiation, and GSK-3, β-catenin, and AKT, which are regulators of the Wnt pathway. Second, the migration and angiogenesis ability of human umbilical vein endothelial cells (HUVECs) inhibited by ZA could be enhanced by treatment with tFNAs. This treatment showed increased expression of VEGF, HIF1α, TGFβ1, IGF, and PDGF. Third, the immune microenvironment is important for curing BRONJ, as regulated by tFNAs. Macrophages can polarize into the M2 phenotype, secreting factors for anti-inflammation and wound healing instead of the M1 phenotype, which induces the secretion of various pro-inflammatory factors. Besides, ROS production in macrophages was also decreased upon exposure to tFNAs. These results provide evidence supporting the excellent therapeutic efficacy of tFNA on BRONJ.

### Cartilage tissue regeneration

Cartilage tissue in the craniofacial region is mainly divided into three parts: the nasal, auricular, and temporomandibular cartilage. While arthritic knees, hips, or shoulders often require cartilage regeneration, it is also important and urgent to promote craniofacial cartilage regeneration. These cartilage tissues are responsible for maintaining the appearance and function of the corresponding regions. Although there are different types of cartilage, such as hyaline cartilage, fibrocartilage, and elastic cartilage, the cartilage components remain similar: chondrocytes, type II collagen (COL-II), and proteoglycans.^163^ Hence, the approaches to enhance cartilage regeneration in the knees, hips, and shoulders are also suitable for craniofacial areas. Cartilage tissue possesses limited self-repair ability in the absence of blood supply and stem cells. Autologous chondrocyte implantation and tissue engineering approaches have succeeded in repairing the cartilage or replacing damaged cartilage. Thus, it is necessary to enhance the proliferation and migration of chondrocytes and adopt advanced nanomaterials for cartilage repair and regeneration.

As previously mentioned, tFNAs exhibit significant therapeutic effects and regenerative abilities and help promote stem cell differentiation, which also has a positive effect on cartilage regeneration. Chondrocytes can quickly and efficiently internalize tFNAs without the help of auxiliary molecules.^99^ Once inside chondrocytes, tFNAs could upregulate the mRNA and protein expression of chondrogenic markers such as COL-II and aggrecan (AGN) to maintain the typical chondrocyte phenotype, accompanied by an upregulation of the mRNA expression of *Notch1*, *Notch3*, and *Hes1*, which are related to the Notch signaling pathway. Chondrocyte proliferation was also facilitated by tFNAs, upregulating the expression of the classical Wnt pathway. Treatment with tFNAs at a concentration of 250 nmol·L^−1^ has the most positive effect on chondrocyte proliferation and phenotype maintenance.^80^ In addition, it was proven that chondrocyte migration was enhanced in the presence of tFNAs at the optimal concentration of 250 nmol·L^−1^. The mechanism underlying this enhanced migration includes increased expressions of Notch pathway-related genes and proteins, such as RhoA, ROCK2, and vinculin.^88^ Furthermore, tFNAs were reported to enhance chondrocyte autophagy through the upregulation of autophagy-related genes and activating the PI3K/AKT/mTOR signaling pathway.^55^ By activating autophagy, chondrocyte apoptosis was inhibited, and oxidative stress was reduced upon exposure to tFNAs (250 nmol·L^−1^) in an OA model, revealing that tFNAs might have the potential for treating OA.^99^ Thus, given the various advantages of the tFNA-induced promotion of chondrocyte proliferation, migration, autophagy, and phenotype maintenance of chondrocyte phenotype, and inhibition of chondrocyte apoptosis in an OA model, tFNAs modified with wogonin (named TWC), a traditional Chinese monomer, were explored to treat OA. It was found that chondrocytes treated with IL-1β absorbed more tFNAs than normal chondrocytes, as confirmed via fluorescence analysis and flow cytometry.^99^ A satisfying result was acquired: TWCs significantly inhibited oxidative stress and effectively suppressed inflammation in vitro. The underlying mechanism was then revealed: the expression of an inflammatory mediator (TNF-α) and matrix metalloproteinases (MMP1, MMP3, and MMP13) were downregulated, while the expression of chondrogenic markers (COL-II and AGN), tissue inhibitor of metalloproteinase 1 (TIMP1), and B-cell lymphoma 2 (BCL2) were upregulated in the presence of TWC. In vivo, TWC notably increased the bone mineral density of regenerated bone tissues, suppressed chondrocyte apoptosis and the expression of inflammatory mediators, and promoted the expression of chondrogenic markers.^60^ Therefore, tFNAs show promise as a nanomaterial for cartilage tissue regeneration applications.

### Neural repair and regeneration

**Fig. 5:**
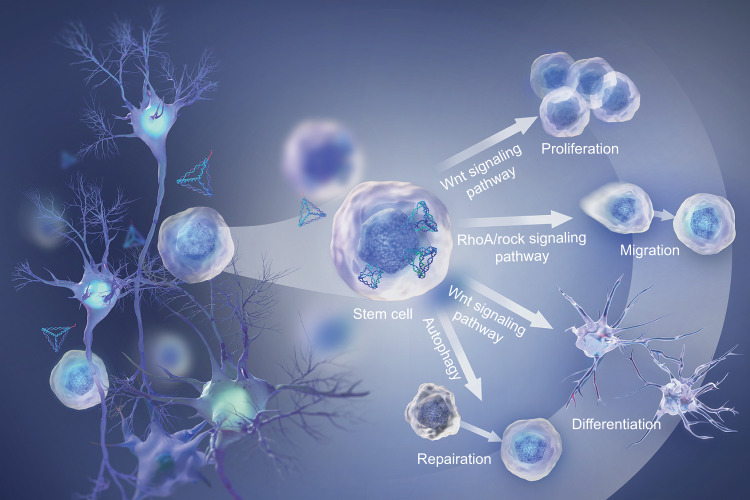
tFNAs enhance nerve repair and regeneration. The proliferation, migration, differentiation, and self-repair abilities of nerve stem cells were improved by treatment with tFNAs

The nervous system is critically important for regulating physiological functions, including those in the craniofacial regions, playing a leading role in all organ systems. Given that neural cells have poor self-renewal and self-repair abilities, damaged nerves are considerably challenging to heal and regenerate.^164,165^ The transplantation stem cells, especially NSCs, are ideal for treating nerve damage, drawing considerable attention in biomedical fields.^166,167^ Although more scientists are doing extensive research in stem cell therapy for nerve repair and regeneration, there remains a significant challenge in effectively enhancing the proliferation, migration, and differentiation of autologous and transplanted NSCs. tFNAs may show considerable promise for promoting nerve repair and regeneration based on our previous studies. As one of the in vitro models of NSCs, neuroectodermal stem cells (NE-4C) possess the ability to proliferate and differentiate into the neuronal lineage.^168,169^ It has previously been shown that tFNAs fluorescently labeled with Cy5 were found in the cytoplasm of NE-4C cells, while ssDNA was not, paving an ideal way for function exertion of tFNAs. After entering cells, tFNAs could promote self-renewal and proliferation by regulating the cell cycle and upregulating the expression of β-catenin, Lef-1, and cyclin-D. An increase in β-III-tubulin expression induced the accelerated differentiation of NE-4C in the presence of tFNAs at a concentration of 250 nmol·L^−1^. Notch-1 has been proven to inhibit the differentiation of stem cells, including Hes-1 and Hes-5. tFNAs can markedly decrease the mRNA and protein expression of Notch-1, Hes-1, and Hes-5 to enhance the differentiation of NE-4C cells.^81^ Subsequently, the migration of NE-4C cells as detected through wound healing and Transwell chamber assays was improved by tFNAs at the same concentration. The promotion of cell migration was due to the upregulated mRNA and protein expression of RHOA, ROCK2, and vinculin upon exposure to tFNAs.^170^ Therefore, since tFNAs enhanced the proliferation, migration, and differentiation of NSCs in a spinal cord injury (SCI) model, tFNAs combined with NSCs exhibited excellent neuroprotection and neuroregeneration properties.^91^ It was observed that the survival rate of transplanted NSCs and nerve conduction function were improved in the NSCs/tFNA-treated group. The number of neurons and oligodendrocytes differentiated from the transplanted NSCs induced by tFNAs remarkably increased in the NSCs/tFNA group compared to the tissues with higher Nestin and myelin basic protein (MBP) expression and lower glial fibrillary acidic protein (GFAP) expression. These results demonstrated that both tFNAs and NSCs display potential neural regeneration and repair effects (Fig. 5).

Nerve regeneration and repair are crucial in various neurodegenerative pathologies (AD and PD), hypoxia-ischemic brain injury (acute ischemic stroke and ICH), and nerve injuries caused by trauma. As one of the most common and most presentative age-related neurodegenerative diseases, AD is mainly characterized by progressive neuronal apoptotic death, leading to memory dysfunction, language impairment, and personality changes, accompanied by cognitive dysfunction, and finally evolving into dementia.^171,172^ An increase in neuronal apoptosis induced by beta-amyloid (Aβ) deposition in the cerebral cortex and hippocampus accelerates the progress of the disease.^173,174^ Inhibiting the apoptosis of neuronal cells is an effective way to treat AD. tFNAs protected injured neuronal cells and suppressed cell apoptosis in an AD cell model (Aβ-induced cytotoxicity in PC12 cells) by changing the abnormal cell cycle by regulating the ERK1/2 signaling pathway.

Meanwhile, tFNAs can also reduce the levels of intracellular ROS and inhibit caspase activity induced by Aβ25−35 to protect neuronal cells from apoptosis.^102^ In vivo experiments confirmed that memory and learning abilities improved in an AD rat model as observed via behavioral test. The Nissl and TUNEL staining results showed that both Aβ25−35 expression and cell apoptosis in the hippocampus were inhibited in the tFNA-treated group.^103^ Since tFNAs might be a potential drug for treating AD, we also applied them to treat another neurodegenerative disease, PD. Cui and her colleagues revealed that the 1-methyl-4-phenyl-1,2,3,6-tetrahydropyridine (MPTP)-induced sPC12 cell apoptosis was greatly reduced and that the AKT/PI3K signaling pathway was activated after tFNA treatment. More importantly, the expression of α-synuclein, a specific biomarker of PD, was reduced, and the genes and proteins related to the mitochondrial apoptotic pathway were changed in the presence of tFNAs.^175^ To enhance the therapeutic efficacy of tFNAs on PD, vitamin B12 (VB12), inhibiting the activity of leucine-rich repeat kinase 2 (LRRK2), a major neurotoxicity factor, was loaded onto tFNAs, named TVC. After penetrating the BBB, TVC can effectively clear the accumulation of abnormal LRRK2 proteins, increase autophagosome formation, and induce autophagic flux by regulating the PI3K/Akt/mTOR signaling pathway.^176^ Besides, Li et al. adopted tFNAs to load a microRNA-22-3p for treating damaged neurons, showing a synergistic therapeutic effect on neuroprotection and neuroregeneration characterized by a higher expression of TrkB and BDNF.^177^ These results suggest that tFNAs exert excellent neuroprotective and neurorestorative properties on neuronal cells and could be modified with drugs or molecules to improve their therapeutic efficacy.

In addition to neurodegenerative diseases, tFNAs have also conferred neuroprotection and neuroregeneration effects in other nervous system disorders. Stroke is caused by an intracranial ischemic or spontaneous hemorrhage. It has high morbidity and mortality and is a global health burden.^178^ tFNAs have been applied in both diseases to explore whether they can positively influence neurological inflammation. Acute ischemic stroke can be established via oxygen-glucose deprivation/reoxygenation treatment using a neuron cell line (SHSY-5Y cells) in vitro. tFNAs effectively reversed the neuronal loss, ameliorated the ischemic hemisphere microenvironment, and alleviated cell apoptosis by inhibiting inflammation and upregulating erythropoietin expression. In vivo rat models of transient middle cerebral artery occlusion (tMCAo), the TLR2-MyD88-NF-κB signaling pathway is involved in the regulation of neurological deficit repair and cell apoptosis inhibition in the presence of tFNAs.^93^ For other types of stroke caused by ICH, Fu et al. used tFNAs to carry siCCR2 (tFNA-siCCR2) to suppress the expression of CCR2 by mediating the recruitment of different kinds of inflammatory and immune cells.^179^ It has been proven that tFNA-siCCR2 improved hematoma absorption, extenuated neurological inflammation by regulating the expression of pro-inflammatory/anti-inflammatory factors, and restored neurological function.

The facial nerve is the seventh pair of brain nerves in the craniomaxillofacial region. It is composed of sensory, motor, and parasympathetic nerve fibers, managing various activities such as taste, facial expression, muscle movement, and controlling the secretion of glands, such as the sublingual, submandibular, and lacrimal glands.^180^ Injured facial nerves essentially affect their function, sometimes leading to limb paralysis. Thus, restoring damaged facial nerves became a research focus in biomedicine. Although SCs can differentiate into repair SCs to protect the neurons and promote axon growth, the restorative ability of repair SCs triggered by injuries is comparatively limited.^181^ Increasing the number of SCs and repairing SCs may be a practical solution. A research report suggested that tFNAs could improve the proliferation and migration of SCs, along with an upregulation of the protein expression of neurotrophins and myelin sheath. In facial nerve crush injury rat models, TNFA treatment restored muscle movement and improved the efficiency of nerve conduction by regulating the NGF/PI3K/AKT signaling pathway related to nerve repair.^90^ These results reveal that tFNAs have neuroprotective properties.

### Vascular regeneration

The vascular system, which is responsible for oxygen and nutrient supply and metabolic output, is a sealed piping system throughout the human body, including the craniomaxillofacial regions. Angiogenesis plays a highly significant role in tissue repair and regeneration and the reconstruction of damaged tissues.^182^ Blood vessel formation in defective and transplanted tissues needs to enhance the proliferation and migration of endogenous ECs and recruit or supplement exogenous cells.^183^ Zhao et al. found that large amounts of tFNAs could enter ECs and promote their proliferation and migration. The tube formation assay, a classic experiment for testing angiogenesis, displayed that the tube junctions, segments, and lengths have been notably improved with higher gene expressions of *VEGF-A* (*-B*, *-R1*, *-R2*), *MMP2/9*, *IGF1*, *PDGF*, and *TGFβ1*. Similar protein expression trends were also observed for VEGF-A/-R2 and MMP2/9 after treatment with tFNAs. They revealed that the Notch signaling pathway is tightly involved in the tFNA-induced regulation and functionalization of ECs and the enhancement of angiogenesis.^85^ The ability of tFNAs to promote angiogenesis has been further improved by Zhao and her colleagues by loading aptamer 02 (Apt02), an alternative to VEGFA, and aptamer VEGF (AptVEGF), enhancing angiogenesis.^86^ Both tFNA−Apt02 and tFNA−AptVEGF showed stronger angiogenesis abilities than pure tFNA by accelerating the proliferation and migration of ECs, vascular tube formation, and spheroid sprouting in vitro and in vivo. In pathological conditions such as diabetic wound healing, tFNAs exhibited an excellent ability for enhancing angiogenesis.^184^ In the diabetic wound healing model induced via AGEs, increased vascularization was observed after stimulation by tFNAs, and the upregulation of VEGF-A expression was observed from the results of the tube-formation assay under inflammatory and oxidative conditions. In addition, Ge et al. indicated that tFNAs connecting with miR-126 (tFNAs-MMs) could enhance the proliferation and migration of healthy HUVECs. Moreover, in the damaged endothelium conditions induced by LPS, tFNAs-MMs inhibited cell apoptosis via downregulation of caspase3 and recovered the angiogenesis ability of impaired HUVECs by decreasing the negative regulators of VEGF (SPRED1 and PIK3R2). tFNAs-MMs showed the excellent properties of both tFNAs and miR-126 in maintaining vascular homeostasis and repairing early-stage vascular damage.^185^

### Skin and mucosa repair

**Fig. 6:**
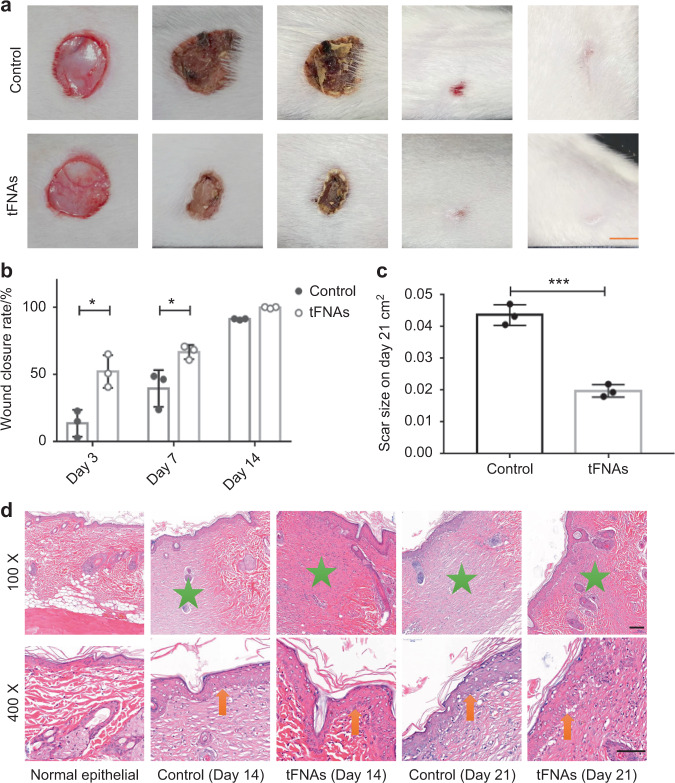
tFNAs promote skin wound healing and decrease scar formation. **a** Photographs of skin wound healing in rats treated with saline or tFNAs. **b**, **c** Statistical analysis of the wound closure rate and scar size. **d** H&E staining of the epidermis. Scale bar: 100 µm. Reproduced with permission.^84^ Copyright ©, 2020 Springer Nature

The skin tissue covers the surface of the human body and forms the first line of defense for the protection of the internal environment from external factors. The functions of the skin include protection, excretion, regulation of body temperature, feeling of external stimuli, and maintaining internal homeostasis.^186,187^ Owing to the vulnerability and location of the skin tissue, it could be easily impaired after wound infection, burns, surgery, trauma, and other skin loss injuries. Recently, scientists improved their understanding of the complicated process and mechanism of cutaneous repair and wound healing. There are four phases in the wound healing process: inflammation, tissue formation, reorganization, and remodeling.^188^ Thus, inhibiting inflammation, promoting the proliferation and migration of fibroblasts, and accelerating the epithelialization are necessary for repairing skin wounds and reducing scar formation. tFNA, a promising DNA nanomaterial for tissue regeneration, was used to explore applications to cutaneous wound healing. It was found that tFNA increased skin wound closure and decreased scar formation in rat cutaneous wound models. The wound healing rate in the tFNA-treated group was highly improved after treatment for 21 days (Fig. 6). Further histopathological staining of epithelial tissue and the hypodermis revealed a smaller scar area, a thicker epidermis, and fewer inflammatory cells in the tFNA-treated group. Additionally, tFNAs at an optimum concentration of 125 nmol·L^−1^ could markedly mitigate skin fibrosis in wounds by downregulating the protein expressions of TNF-α and IL-1β. In in vitro experiments, the AKT signaling pathway was confirmed to regulate tFNAs, enhancing the proliferation and migration of keratinocytes (HaCaT cells) and fibroblasts (HSF cells), along with a notably increased secretion of VEGF and bFGF in HSF cells and a decreased production of TNF-α and IL-1β in HaCaT cells.^84^ Interestingly, tFNAs can also clear senescent human dermal fibroblasts (SEN-HDFs) by regulating apoptosis-relevant signaling pathways and increasing cytochrome C expression.^189^

Compared to skin wounds, wounds in the oral mucosa show accelerated healing and minimal scarring.^190^ However, wound location, size, and exceptional internal environment affect soft tissue repair,^191^ especially in patients with diabetes.^192^ People with diabetes with high blood glucose levels could experience chronic damage and dysfunction to most of their organs.^193^ Among them, diabetic foot ulcerations (DFUs) can delay the wound healing process due to a decrease in angiogenesis and an increase in the expressions of inflammatory factors.^194^ Facilitating angiogenesis, inhibiting inflammation, promoting cell proliferation and migration, and suppressing oxidation are deemed to extenuate diabetes-mediated oral mucosa wounds. Therefore, Lin et al. adopted tFNAs to treat mucosa wounds in diabetes induced by advanced glycation end products (AGEs).^184^ Not surprisingly, a vessel-like structure was found in the tFNA-treated group, coupled with a remarkable increase in endothelial cell proliferation and migration and higher VEGF-A expression. In addition, tFNAs mitigated the inflammatory reactions in the mucosa wounds by decreasing ROS levels and upregulating the expressions of Akt, Nrf2, and HO-1. tFNAs effectively accelerated the mucosa wound healing in a diabetic rat model (Fig. S3a, b). Histological staining (HE and Masson) and CD34 immunohistochemistry of buccal mucosa sections on the 14^th^ day revealed smaller gap lengths in the epidermal layer, fewer inflammatory cells, thicker epidermal layer, and more neovascularization in the tFNA-treated groups than those in the diabetic control group (Fig. S3c–e). Taken together, tFNAs play a positive role in diabetic mucosa wound healing.

### Muscle regeneration

The muscle in the craniomaxillofacial regions is mainly divided into masticatory and facial muscles, which are markedly responsible for mastication and facial expression. If these muscles are damaged or deteriorated, their normal functions cannot be completed. The stemness and self-renewal of myoblasts are considerably important for muscle tissue regeneration to treat degenerative muscle diseases or traumatic muscle injury.^195^ Due to the limited regenerative abilities of muscle tissue in the body, maintaining the stemness, enhancing self-renewal rates of myoblasts, and promoting cells migration were considered to be significantly feasible methods for curing diseases resulting from muscle damage.^196^ Not surprisingly, tFNAs showed a remarkable promotion effect on the proliferation and migration of most mammalian cells, similarly applied to myoblasts.^197^ C2C12 cells, a representative myoblast cell line used in in vitro experiments, can noticeably internalize large amounts of tFNAs with the help of any delivery molecules. Benefiting from the tFNAs in a concentration-dependent manner, the proliferation of C2C12 cells was improved, as revealed from the CCK8 and EdU staining assays. The Wnt signaling pathway was also detected to determine the mRNA and protein expressions of the molecules in that pathway in C2C12 cells after treatment with tFNAs. We observed a markedly decreased expression of GSK3-β and prominently increased expressions of β-catenin, Lef-1, and Cyclin-D1. tFNAs have also enhanced autophagy and are critical to the self-renewal of myoblasts through the promotion of the expressions of beclin1 and LC3 in C2C12 cells. With the inhibition of paired box 7 (PAX7), tFNAs showed the potential to maintain the stemness of C2C12 cells. In acute muscle injury mouse models, a gradual decrease in PAX7-positive cells was observed in the tFNA-treated group in the healing process from 3, 7, and 14 days. Additionally, the results of HE and Masson staining suggested that tFNAs could increase the number of myoblasts and hasten the repair of damaged muscle. Combined with the above results, tFNAs conferred a promising potential regenerative ability in muscle tissue.

### Multi-tissue integrative regeneration

Periodontitis is a common chronic inflammatory disease induced by various factors such as microbes, genetics, environment, and other factors impairing the supporting structures of the teeth (gingiva, periodontal membrane, periodontal ligament, alveolar bone, and cementum), causing periodontal bone resorption.^198,199^ However, a period of chronic inflammation persisting without treatment leads to teeth looseness and loss, interfering with dentition functions (chewing, talking, and facial aesthetics), and influencing the patients’ quality of life.^200^ Periodontitis has a high prevalence in adults over the age of 35, affecting approximately 50% of people worldwide, with severe periodontitis cases accounting for approximately 12% of the world’s population.^201^ Numerous inflammatory factors and excessive oxygen-free radicals secreted by inflammatory cells such as polymorphonuclear lymphocytes can lead to or worsen the mild inflammation associated with multiple systemic diseases (diabetes, arterial endothelial dysfunction, rheumatoid arthritis, chronic kidney diseases, respiratory disease complications, or cancer).^200,202^ Hence, periodontitis prevention and treatment are deemed essential for general human health. Apart from eliminating biofilm microbiota and controlling inflammation, promoting periodontal tissue regeneration (particularly cementum regeneration and alveolar bone regeneration) is also significant for curing periodontitis.^203^

#### Anti-bacterial and anti-inflammation

With the abuse or misuse of conventional antibiotics, multiple-drug resistant (MDR) strains have become more common, seriously affecting human health, putting enormous pressure on the global public health system.^204^ Searching for novel anti-bacterial drugs or enhancing the therapeutic efficacy of conventional antibiotics is the best way to solve antibiotic resistance. As a result, the ability of pathogenic bacteria to resist common and conventional antibiotics, such as erythromycin and ampicillin, have increasingly improved every year.^205^ A marked decrease in membrane permeability, affinity, and efflux pump increase leads to a lower drug concentration in cells. Therefore, Sun et al. first adopted a novel delivery vehicle to help drug uptake and accumulation and address this pressing problem.^206,207^ Both erythromycin and ampicillin successfully loaded onto tFNAs could be efficiently transported into *Escherichia coli* (E. coli)^206^ and MRSA.^207^ A deeper investigation found that tFNAs as carriers may reduce the destabilization of the bacterial cell membrane and increase membrane permeability, allowing drugs to move across the thick membrane, as evidenced by the results of the o-Nitrophenyl-β-D-Glucopyranosides (ONPG) test and the measurement of intracellular [K^+^] and [Na^+^]. These complexes have a better affinity against bacterial resistance to antibiotics, lower levels of antibiotic resistance, and stronger antibacterial effects than free antibiotics. The molecular mechanism of tFNAs-ampicillin against MRSA demonstrated that the mRNA expressions of *murA and murZ* responsible for bacterial membrane formation are downregulated, and that of *PBP2* related to the management of antibiotic sensibility is upregulated. The combination of tFNAs and antibiotics provides a promising platform for the widespread use of conventional antibiotics even with bacterial resistance.

In addition to traditional antibiotics, there are two novel antibacterial drugs, named antimicrobial peptides (AMPs) and asPNAs. AMPs are short cationic peptides that can specifically bind to bacterial cell membranes through electrostatic absorption to destroy the membrane morphology and kill bacteria.^208–210^ A new delivery vehicle urgently needs to be constructed to prevent proteases from degrading AMPs and enhance the effects of AMP against antibiotic-resistant bacteria. For example, tFNAs with a negative charge and powerful cell-entry performance could interact with the cationic peptide GL13K according to electrostatic reactions at proper ratios via a simple approach.^211^ The positively charged tFNAs-GL13K complexes (TGCs) showed stronger red fluorescence signals than free GL13K in *E. coli* and *P. gingivalis* cytoplasms.

Furthermore, TGCs seriously deformed both *E. coli* and *P. gingivalis* membranes, showing shrinkage, pore formation, debris, and fracture compared to treatment with GL13K alone. Besides, the antibacterial activity of TGCs was also detected by measuring its optical density at 600 nm (OD_600_) and via live/dead bacterial staining analysis. Our results demonstrated that TGCs effectively increased the death rate of *E. coli* and *P. gingivalis*. Apart from natural peptide AMPs, there are also synthetic DNA analog short peptides called asPNAs that aim to suppress microbial gene expression via complementary base pairing. Although asPNAs have strong stability, affinity, and enzyme degradation resistance, their lower cell absorption rate limits their applications. Considering the rules of Watson-Crick, a 12-mer asPNA specifically targeting the gene *ftsZ* was paired with a region of a single strand of tFNAs; thus, the intercalation of asPNAs into tFNAs maintained the structure, size, and properties of the vehicle.^126^ It has been proven that tFNA-asPNA complexes (TPCs) were successfully constructed with high efficiency and could be transfected into MRSA cells in large amounts. The MRSA cells’ activity was measured by observing culture turbidity, OD_600_ value, and the growth curves after treatment with different concentrations of TPCs. The results revealed that the inhibition effect of TPCs on MRSA increased in a concentration-dependent manner. Moreover, the gene expression of *ftsZ* also decreased with an increased concentration of TPCs, suggesting that *ftsZ* is tightly involved in TPCs suppressing the activity of MRSA.

**Fig. 7:**
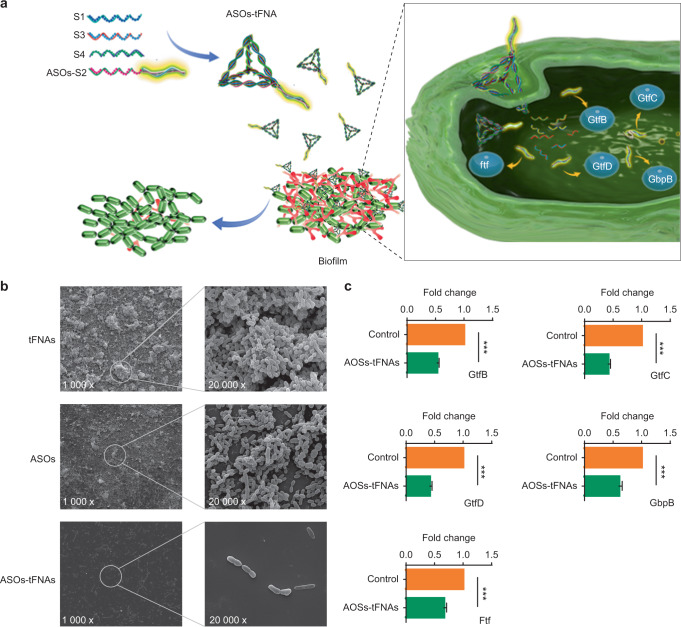
tFNAs-ASOs inhibit the formation of bacterial biofilms. **a** Schematic representation of the fabrication of tFNAs-ASOs, and the tFNA-ASO-mediated suppression of bacterial biofilm formation via the regulation of biofilm-related genes. **b** SEM images reveal the architecture of biofilms after treatment with tFNAs, ASOs, and tFNAs-ASOs. **c** The levels of targeted EPS synthesis-related genes were examined using qPCR. Reproduced with permission.^57^ Copyright ©, 2020 Springer Nature

Treating periodontitis requires the elimination of biofilm plaques that form on the tooth surface. However, there are several difficulties in eradicating biofilm-associated microbiota, including the presence of the periodontal pocket, bacteria with antibiotic resistance, and the non-targeted overuse of drugs upon oral administration.^212^ Bacteria in biofilms possess a stronger resistance to antibiotics compared with planktonic ones. Thus, Zhang et al. developed an antisense nucleotide sequence targeting various specific genes and proteins to inhibit extracellular polysaccharides (EPS), providing adhesion, toxicity, and resistance for biofilms. tFNAs carried multi-targeted ASOs to penetrate the membranes of bacteria (*S. mutans*) (Fig. 7a). As expected, the ASO-tFNA complexes were successfully formed. Effective endocytosis could prevent the synthesis of bacterial biofilms by decreasing the expression of target genes such as *gtfBCD*, *gbpB*, and *ftf* without affecting the expression of non-target genes (such as 16 S rRNA) (Fig. 7b, c). This report provided a promising ASO delivery system for treating periodontitis by suppressing early biofilm synthesis.^207^

Controlling inflammation for treating periodontitis is as crucial as controlling bacterial growth. Inflammatory factors in the oral microenvironment could impair the periodontal tissue and bone. A co-culture of PDLSCs and LPS was used to construct an in vitro periodontitis cell model, while pure tFNAs were used to explore their antioxidant and anti-inflammatory effects on periodontitis.^82^ Higher levels of ROS produced by hyperoxidative cells can destroy membranes, cause inflammatory reactions, induce protein changes, and damage cell function. As Zhang et al. reported, tFNAs could notably decrease ROS production stimulated by LPS, but they could not affect ROS production without LPS treatment.^52^ An ROS Assay Kit was then used to analyze whether tFNAs can reduce ROS release in PDLSCs triggered by LPS. Intracellular ROS levels in the tFNA-treated group were lower than that in the control group. The expression levels of inflammatory factors can represent the intensity of inflammation. The expression of pro-inflammatory cytokines TNF-α, IL-6, and IL-1β were significantly reduced in PDLSCs treated with LPS and then with tFNAs. These results suggested that tFNAs could protect PDLSCs against inflammation by decreasing the expression of pro-inflammatory cytokines and reducing ROS production.^82^

#### Promotion of multi-tissue regeneration

**Fig. 8:**
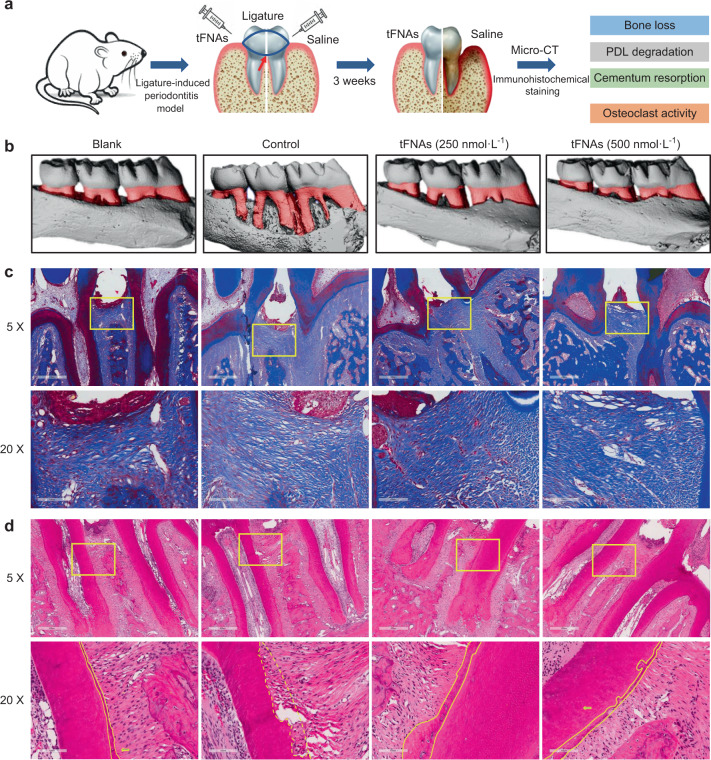
tFNAs protect alveolar bone and periodontal tissues from periodontitis. **a** Schematic diagram of the rat periodontitis experiment. **b** Representative images of micro-CT 3D reconstruction of the left maxillary alveolar bone. **c** Masson’s trichrome staining of periodontal tissues from the different groups. **d** H&E staining of periodontal tissues from the different groups. Reproduced with permission.^82^ Copyright ©, 2021 Elsevier

Pathogenic bacteria and inflammatory reactions can severely damage periodontal tissue homeostasis. Moreover, they could destroy periodontal tissue and bone structures in periodontitis. Hence, repairing damaged tissue and promoting tissue regeneration during periodontitis are also important, in addition to combating bacterial infections and controlling inflammation. DPSCs have been stimulated to proliferate and differentiate into osteo/odontogenic cells upon exposure to tFNAs at a concentration of 250 nmol·L^−1^, as reported by Zhou et al.^95^ They further studied the effects of tFNAs on PDLSC proliferation and osteogenic differentiation. They found that tFNAs with the same concentration could activate the Wnt/β-catenin signaling pathway and accelerate the proliferation and differentiation of PDLSC cells under normal circumstances.^159^ Due to the positive effects of tFNAs on PDLSCs, Zhou and her colleagues verified whether tFNAs had the same effects on PDLSCs under inflammatory conditions. Somewhat expectedly, the migration and osteogenic differentiation of PDLSCs were enhanced in vitro to promote new bone formation in the presence of tFNAs in an inflammatory environment, accompanied by higher expressions of RUNX2 and OPN. In ligature-induced periodontitis rat models, HE staining of the alveolar bone revealed that tFNAs could reduce inflammatory cell infiltration. Additionally, as shown in the immunohistochemical staining of IL-6 and IL-1β, the levels of pro-inflammation factors in the tFNA-treated groups (250 nmol·L^−1^ and 500 nmol·L^−1^) were lower than that in the control group, suggesting that tFNAs suppressed inflammation to decrease the destruction of periodontal tissue during periodontitis. Furthermore, the periodontal ligament matrix and collagen fibers increased, and the number of osteoblasts was significantly decreased. These results mean a decrease in bone resorption after treatment with tFNAs for three weeks, exhibiting that tFNAs protect periodontal tissue and promote their regeneration in inflammatory conditions (Fig. 8).^82^ As a result, tFNAs may also be potential agents for preventive and therapeutic periodontitis.

### Anti-cancer therapy

Cancer, the second leading cause of death worldwide, is characterized by the abnormal proliferation, migration, and differentiation of normal cells. Cancer can occur anywhere in the human body, including the craniomaxillofacial regions. Over the past few decades, although conventional anticancer therapies (surgery, radiation, and chemotherapy) have progressed dramatically, they still have several limitations. With the rapid development of DNA nanotechnology, most barriers in cancer therapies have been effectively removed. We believe that tFNA is the perfect delivery vehicle for targeted molecules and cancer drugs. As previously suggested by Meng et al., Dz13 cleaving the c-Jun mRNA in the cytoplasm, a DNAzyme that has poor stability and low potential for cellular endocytosis, has been loaded into tFNAs. The successful synthesis of tFNAs-Dz13 exhibited exceptional cell entry efficiency and potent inhibition of the growth of human epidermoid carcinoma cells (A431 cells) via silencing of the c-Jun gene.^64^ Another small molecule silencing gene expression is small interfering RNA (siRNA). siRNAs knock down the expression of target genes and have been widely used to kill carcinoma cells. For example, Braf gene mutations induced the occurrence of malignant melanoma. Xiao and his colleagues took the sticky ends connection method to combine tFNAs and a siRNA of Braf (siBraf). AS1411 aptamers were also linked with the tFNAs to target nucleolin expression on the surfaces of A375 cells. Decreasing the mRNA and protein expression of Braf and tFNAs-AS1411-siBraf apparently downregulated the levels of phosphorylated MEK and ERK, as observed from the results of immunofluorescence detection and western blotting.^59^ In addition to directly killing cancer cells, blocking oxygen and nutrient uptake is another important way to inhibit angiogenesis. VEGF regulates endothelial cell survival, growth, and migration, which are crucial for angiogenesis. Pegaptanib can target the inhibition of VEGF and could be a potential anticancer drug. To improve cell entry efficiency and biostability in vivo, tFNAs were introduced to be pegaptanib nanocarriers. The stability in the serum and cell binding capacity of pegaptanib was improved after combining with tFNAs. Meanwhile, cell viability analysis revealed that pegaptanib-tFNAs at a concentration of 375 nmol·L^−1^ could kill 45% of HUVECs and 26% of oral squamous carcinoma cells (Cal 27). Moreover, pegaptanib-tFNAs can markedly suppress the migration and tube formation of HUVECs stimulated by VEGF.^213^ Apart from these aforementioned anticancer drugs, there are still various molecules that could inhibit tumors, including PTX,^85^ DOX,^214^ 5-FU,^215^ microRNAs,^184^ and tumor-penetrating peptides^216^ loaded into tFNAs to further their lethality. We believe that tFNAs are a promising delivery vehicle for various anticancer drugs.

### Treatment of immune-associated diseases

Sjögren’s syndrome (SjS), extensively characterized by the damage of exocrine secretory glands such as the salivary gland and lacrimal gland and lymphocytic infiltration, is typically considered a complicated chronic systemic autoimmune disease. In serious cases, SjS may lead to the destruction of organ systems or B-cell lymphoma. The hallmark feature of SjS is the salivary gland damage mediated by immune cells.^217^ SjS is a global disease with a prevalence of 0.3%~0.7% in China and a higher incidence in the elderly population, at approximately 4%. The female incidence rate is notably higher than that in males.^218^ However, traditional treatments have focused on controlling the symptoms but have not addressed the underlying causes of inflammatory reactions. CD4^+^ Foxp3^+^ regulatory T (Treg) cells play an indispensable role in maintaining immune tolerance to self-antigens by inhibiting the activation of immune cells. Accounting for the anti-inflammatory and immunomodulatory ability of tFNAs, as reported by Zhang et al.,^52^ 3D DNA nanomaterials may be used to prevent and treat immune tolerance-associated diseases. Following this, Gao and her colleagues utilized a non-obese diabetic (NOD) mice model to test the immunomodulation capability of tFNAs.^219^ The male NOD mouse is a well-established and representative animal model for SjS occurring spontaneously with excessive autoimmunity.^220^ In prediabetic NOD mice, tFNA treatment at a concentration of 250 nmol·L^−1^ can lead to immune tolerance by inhibiting diabetogenic T cell proliferation and increasing the proportion of regulatory T cells. Furthermore, a mechanistic study found that STAT signals are tightly involved in regulating immune tolerance induced by tFNAs. In detail, STAT5 overexpression induced Tregs to protect against T1D, while the expression of STAT3 that damages regulatory T cells and upregulates the proportion of Th17 cells is sharply suppressed in the tFNA-treated group. Furthermore, we also found that tFNAs significantly decreased the levels of p-STAT1 as detected via Phosflow analysis.^219^ As a result, tFNAs may be an immune regulator to prevent the onset of T1D in NOD mice and maintain immune homeostasis.

## Conclusion and prospect

In this progress report, unprecedented advances in self-assembled tFNAs have been made, focusing on applications in regeneration medicine. With well-defined synthesis protocols and high programmability, tFNAs were first successfully fabricated by Turberfield and his groups. In the next few decades, researchers have studied the properties and biological characteristics of their specific structures. As previous studies suggested several methods to synthesize tFNAs, the one-step annealing from four particular ssDNAs was undoubtedly the optimal approach, considering the financial and time requirements and yield. tFNAs also possess many attractive features. The most surprising and exceptional characteristic of tFNAs is their ability for cellular internalization and tissue penetration, allowing wide applications in the biomedical field. Compared with ssDNA, many tFNAs could be uptaken into most mammalian cells (stem cells, carcinoma cells, chondrocytes, macrophages, L929, among others) via a caveolin-mediated endocytosis pathway. After entering cells, tFNAs could significantly affect cellular behaviors, including proliferation, migration, autophagy, and differentiation, and can inhibit inflammation, oxidation, and apoptosis. We believe that these two characteristics provide tFNAs the potential for applications in tissue regeneration (bone/cartilage/nerve/skin/vascular/muscle regeneration) and disease treatment (bone defects, neurological disorders, joint-related inflammatory diseases, periodontitis, immune diseases, among others). In addition, the capability of tFNAs to penetrate the skin was demonstrated by Fan’s group, with penetration depths increasing with a decrease in tFNA size. In their setups, 17-nm tFNAs reached the deepest part of the skin (350 µm from the surface), showing the best penetration. This feature is considerably conducive to the transdermal administration of tFNAs. Last but not least, the base complementary pairing principle proposed by Watson and Crick makes tFNAs programmable and modifiable. Nucleic acids and therapeutic molecules can be loaded into tFNAs through sequences extended, sticky-end hybridization, intercalation, and encapsulation according to the intended application and carrying capacity. This excellent characteristic further expands the application range of static forms of tFNAs in various diseases.

With the emergence of stimulus-responsive drug delivery, DNA nanostructures have been fabricated and modified depending on various responsive molecular recognition properties to be carried out and released in targeted areas. A tFNA cage could wrap therapeutic drugs (cytochrome C, RNase A, and melittin) away from enzymatic degradation and unsuitable pH situation, releasing them in targeted regions through various external stimuli. tFNA has an excellent 3D structure with a reversible conformation for controlling drug encapsulation and release. Besides, tFNAs might also serve as the basic structural unit for constructing DNA hydrogels due to their excellent stability and programmability. However, massive efforts are urgently needed to synthesize injectable tFNA hydrogels for more effective tissue regeneration via a sustained release mechanism. In the future, we will further extend the biomedical applications of tFNAs mainly from these two directions. Meanwhile, tFNAs may bring new opportunities, insights, and hopes for disease treatment and tissue regeneration based on their excellent characteristics revealed in previous studies.

## Supplementary information

Supplementary material

Table S1

Figure S1

Figure S2

Figure S3

Copyright file of Fig 4

Copyright file of Fig 6

Copyright file of Fig 7

Copyright file of Fig 8

Copyright file of Fig S3

## Data Availability

Data openly available in a public repository.
