# Supplementary material for: Advances in regenerative medicine applications of tetrahedral framework nucleic acid-based nanomaterials: an expert consensus recommendation
Source: Int J Oral Sci. 2022 Oct 31;14:51. doi: 10.1038/s41368-022-00199-9 (PMC9622686; doi:10.1038/s41368-022-00199-9)

Advances in Regenerative Medicine Applications of Tetrahedral Framework Nucleic Acid-based Nanomaterials: An Expert Consensus Recommendation

Yunfeng Lin^1^*, Qian Li^2^, Lihua Wang^3^, Quanyi Guo^4^, Shuyun Liu^4^, Shihui Zhu^5^, Yu Sun^5^, Yujiang Fan^6^, Yong Sun^7^, Haihang Li^8^, Xudong Tian^8^, Delun Luo^9^, Sirong Shi^1^*

^1^ State Key Laboratory of Oral Diseases & National Clinical Research Center for Oral Diseases & Department of Oral and Maxillofacial Surgery, West China Hospital of Stomatology, Sichuan University, Chengdu, China.

^2^ School of Chemistry and Chemical Engineering, Frontiers Science Center for Transformative Molecules, Institute of Translational Medicine, Shanghai Jiao Tong University, Shanghai, China

^3^ The Interdisciplinary Research Center, Shanghai Advanced Research Institute, Chinese Academy of Sciences, Zhangjiang Laboratory, Shanghai, China

^4^ Institute of Orthopedics, Chinese PLA General Hospital, Beijing Key Laboratory of Regenerative Medicine in Orthopedics, Key Laboratory of Musculoskeletal Trauma & War Injuries PLA, Beijing, China

^5^ Department of Burn Surgery, The First Affiliated Hospital of Naval Medical University, Shanghai, China

^6^ National Engineering Research Center for Biomaterials, Sichuan University, Chengdu, China

^7^ College of Biomedical Engineering, Sichuan University, Chengdu, China

^8^ Jiangsu Trautec Medical Technology Company Limited, Changzhou, China

^9^ Chengdu Jingrunze Gene Technology Company Limited, Chengdu, China

***CORRESPONDING AUTHOR:**

Yunfeng Lin: [yunfenglin@scu.edu.cn](mailto:yunfenglin@scu.edu.cn)

Sirong Shi: sirongshi@scu.edu.cn

State Key Laboratory of Oral Diseases & National Clinical Research Center for Oral Diseases & Department of Oral and Maxillofacial Surgery, West China Hospital of Stomatology, Sichuan University, Chengdu 610041, P. R. CHINA;

Tel: +86-28-85503487; Fax: +86-28-85582167;

**Running title: tFNAs for Regenerative Medicine**

**Table S1.** The sequence of four specific ssDNAs designed for the formation of tFNAs.

| DNA | Sequence |
| --- | --- |
| S1 | 5’-ATTTATCACCCGCCATAGTAGACGTATCACC |
|  | AGGCAGTTGAGACGAACATTCCTAAGTCTGAA-3’; |
| S2 | 5’-ACATGCGAGGGTCCAATACCGACGATTACA |
|  | GCTTGCTACACGATTCAGACTTAGGAATGTTCG-3’; |
| S3 | 5’-ACTACTATGGCGGGTGATAAAACGTGTAGCA |
|  | AGCTGTAATCGACGGGAAGAGCATGCCCATCC-3’; |
| S4 | 5’-ACGGTATTGGACCCTCGCATGACTCAACTGC |
|  | CTGGTGATACGAGGATGGGCATGCTCTTCCCG-3’; |

**Figure S1. tFNA-21, fabricated by one-step annealing, possesses better properties than the tFNAs of other sizes.** (a) One-step annealing approach for the synthesis of tFNAs. (b) 3-arm-junction hybridization method for self-assembled tFNAs. (c) Scaffold-folding approach for the fabrication of tFNAs. (d) Six tFNAs of different sizes (tFNA-7, tFNA-13, tFNA-17, tFNA-21, tFNA-26, tFNA-37). (e) Cellular internalization of tFNAs with different sizes. (f) Cell viability after treatment with differently sized tFNAs. (g) Cell migration was enhanced by differently sized tFNAs. Statistical analysis (n ≥ 3): * compared with control, ^*^P < 0.05, ^***^P < 0.001; # compared with tFNA-7, ^#^P < 0.05, ^##^P < 0.01; $ compared with tFNA-13, ^$^P < 0.05, ^$$^P < 0.01, ^$$$^P < 0.001; & compared with tFNA-17, ^&^P < 0.05, ^&&&^P < 0.001; a compared with tFNA-26, ^a^P < 0.05.


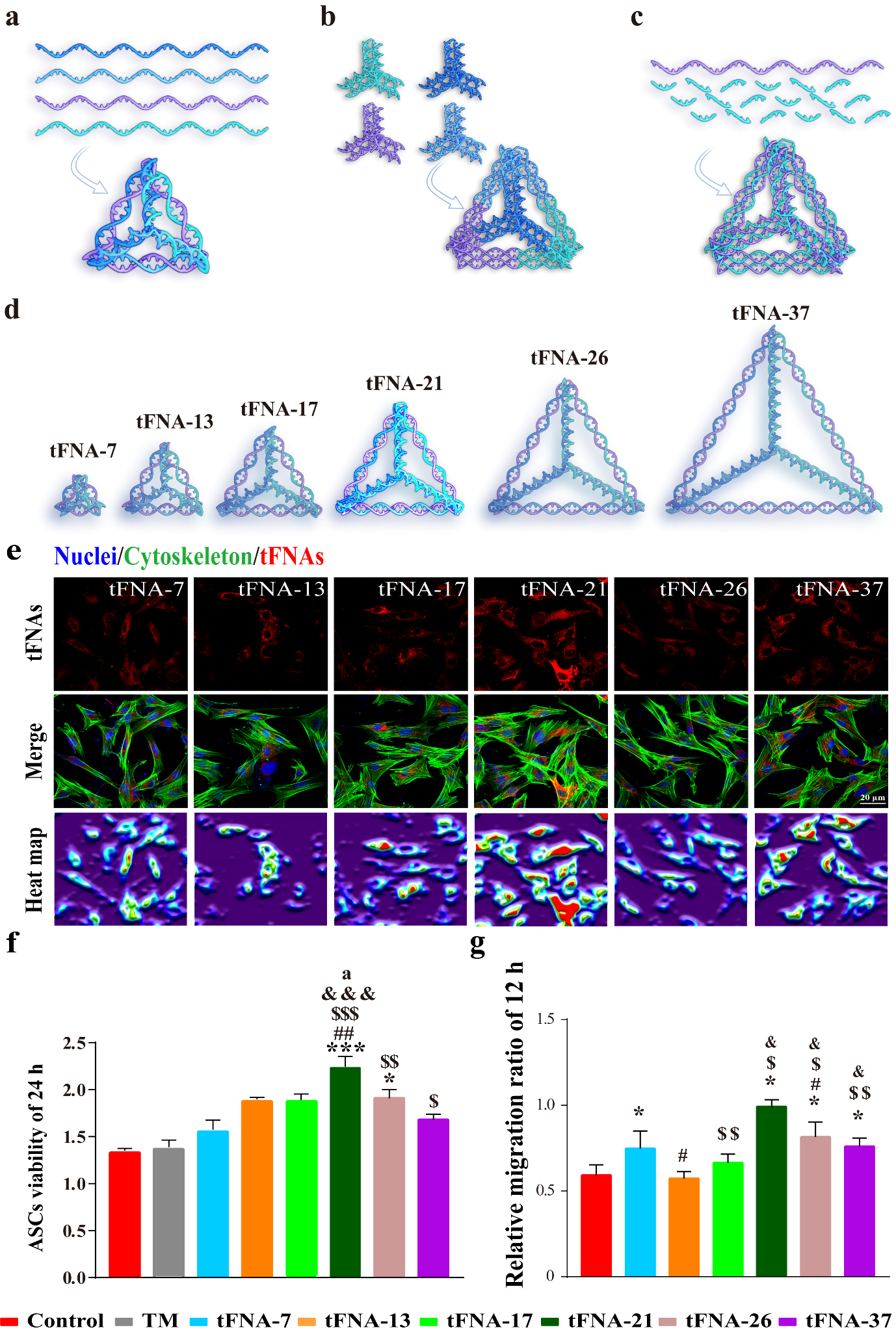


**Figure S2. tFNA functionalization via multiple programmabilities.** (a) Sequences extension type. (b) Sticky-end hybridization type. (c) Intercalation type. (d) Encapsulated type.


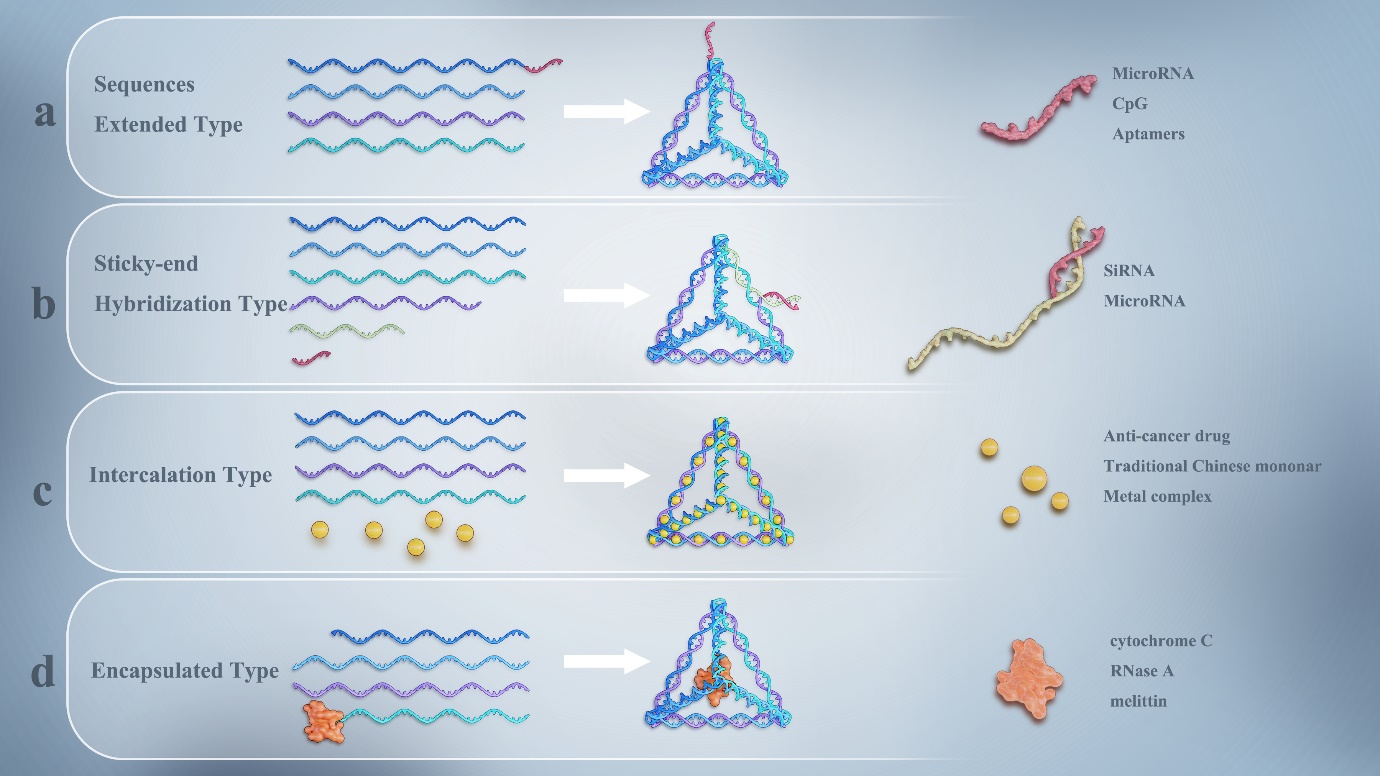


**Figure S3. tFNAs facilitate diabetic mucosal wound healing.** (a) Photographs of mucosal wounds in rats treated with saline and tFNAs. (b) Areas (%) of wound-healing regions in different groups at different time points. (c, d) H&E staining and Masson’s trichrome staining of buccal mucosa sections from diabetic rats treated with saline and tFNAs. (e) Immunohistochemical staining for evaluating CD34 expression. Reproduced with permission.^[185]^ Copyright ©, 2020 American Chemical Society.


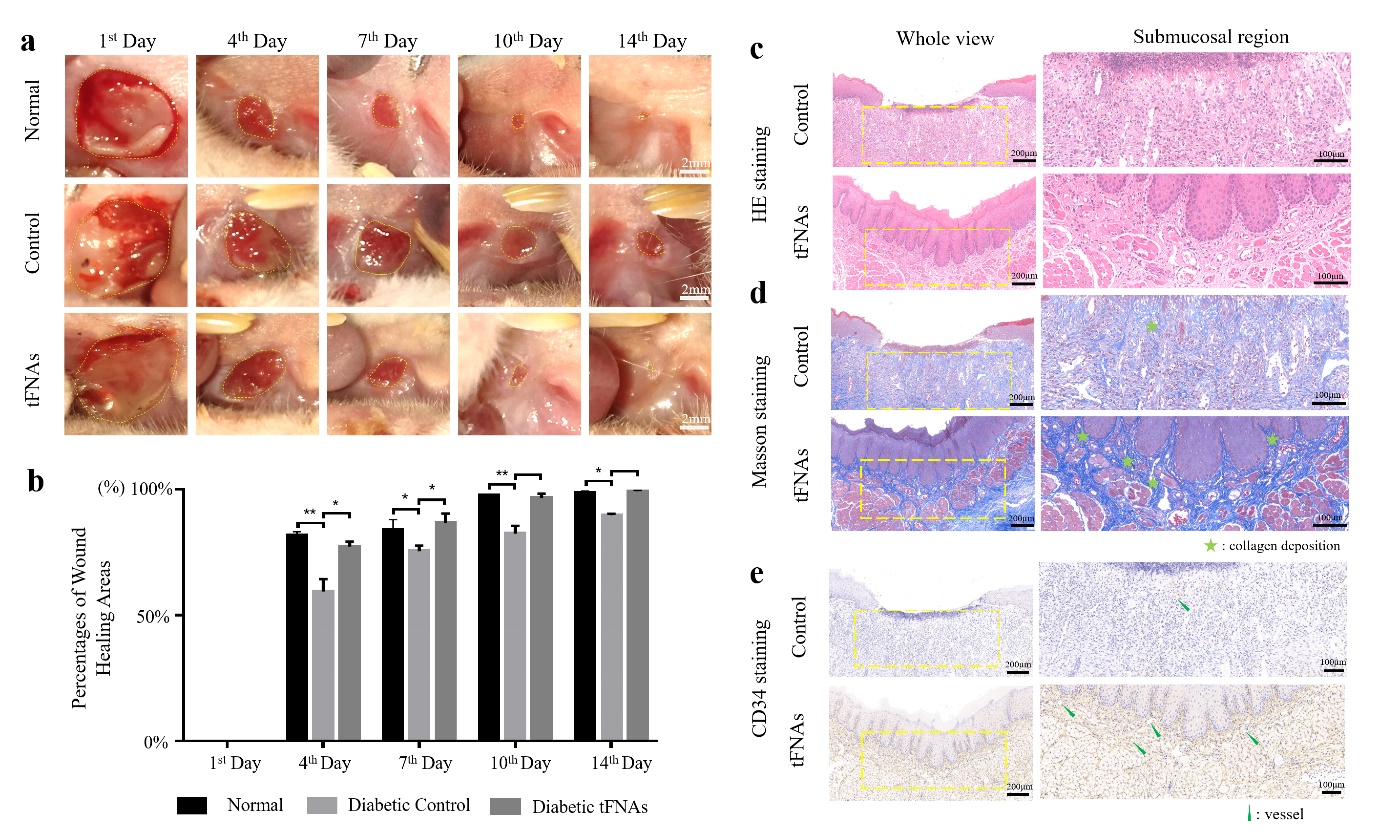

Supplement: Supplementary file 1 — Supplementary material [file 41368_2022_199_MOESM1_ESM.docx]
