# Supplementary material for: Advances in regenerative medicine applications of tetrahedral framework nucleic acid-based nanomaterials: an expert consensus recommendation
Source: Int J Oral Sci. 2022 Oct 31;14:51. doi: 10.1038/s41368-022-00199-9 (PMC9622686; doi:10.1038/s41368-022-00199-9)
Supplement: Supplementary file 2 — Table S1 [file 41368_2022_199_MOESM2_ESM.docx]

**Table S1.** The sequence of four specific ssDNAs designed for the formation of tFNAs.

| DNA | Sequence |
| --- | --- |
| S1 | 5’-ATTTATCACCCGCCATAGTAGACGTATCACC |
|  | AGGCAGTTGAGACGAACATTCCTAAGTCTGAA-3’; |
| S2 | 5’-ACATGCGAGGGTCCAATACCGACGATTACA |
|  | GCTTGCTACACGATTCAGACTTAGGAATGTTCG-3’; |
| S3 | 5’-ACTACTATGGCGGGTGATAAAACGTGTAGCA |
|  | AGCTGTAATCGACGGGAAGAGCATGCCCATCC-3’; |
| S4 | 5’-ACGGTATTGGACCCTCGCATGACTCAACTGC |
|  | CTGGTGATACGAGGATGGGCATGCTCTTCCCG-3’; |
