# Supplementary figures and images for: Advances in regenerative medicine applications of tetrahedral framework nucleic acid-based nanomaterials: an expert consensus recommendation
Source: Int J Oral Sci. 2022 Oct 31;14:51. doi: 10.1038/s41368-022-00199-9 (PMC9622686; doi:10.1038/s41368-022-00199-9)

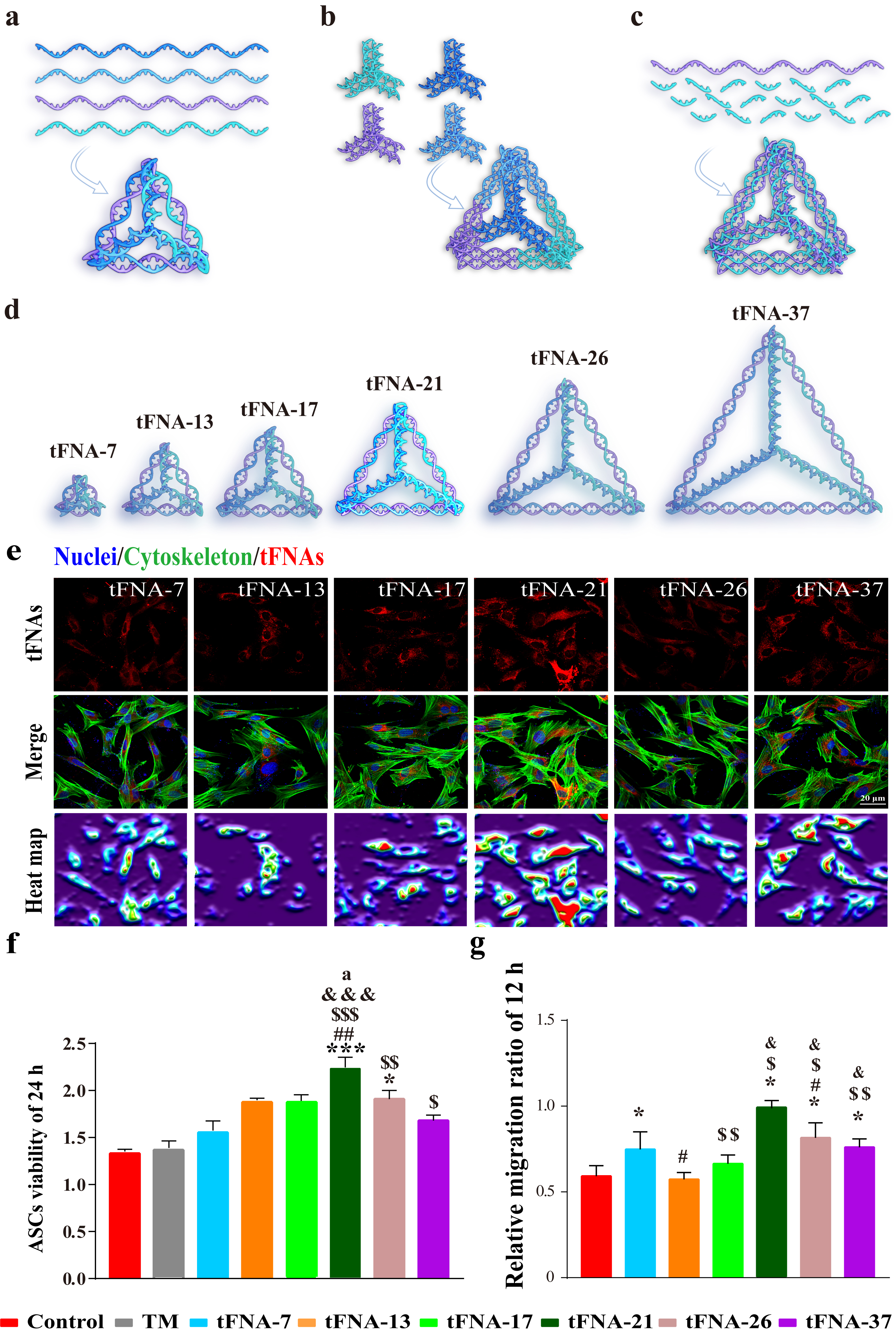

Supplement: Supplementary file 3 — Figure S1 [file 41368_2022_199_MOESM3_ESM.jpg]

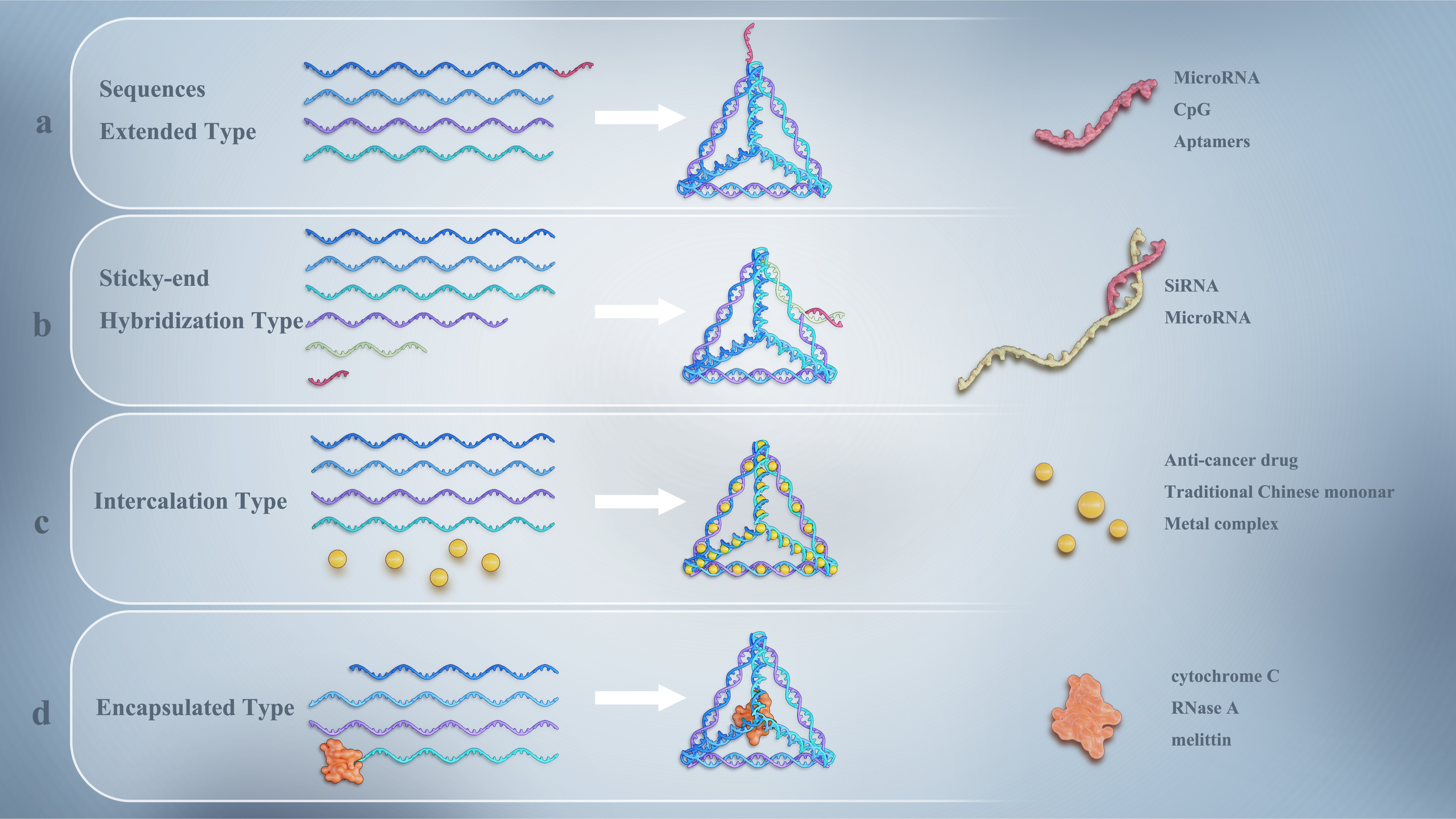

Supplement: Supplementary file 4 — Figure S2 [file 41368_2022_199_MOESM4_ESM.jpg]

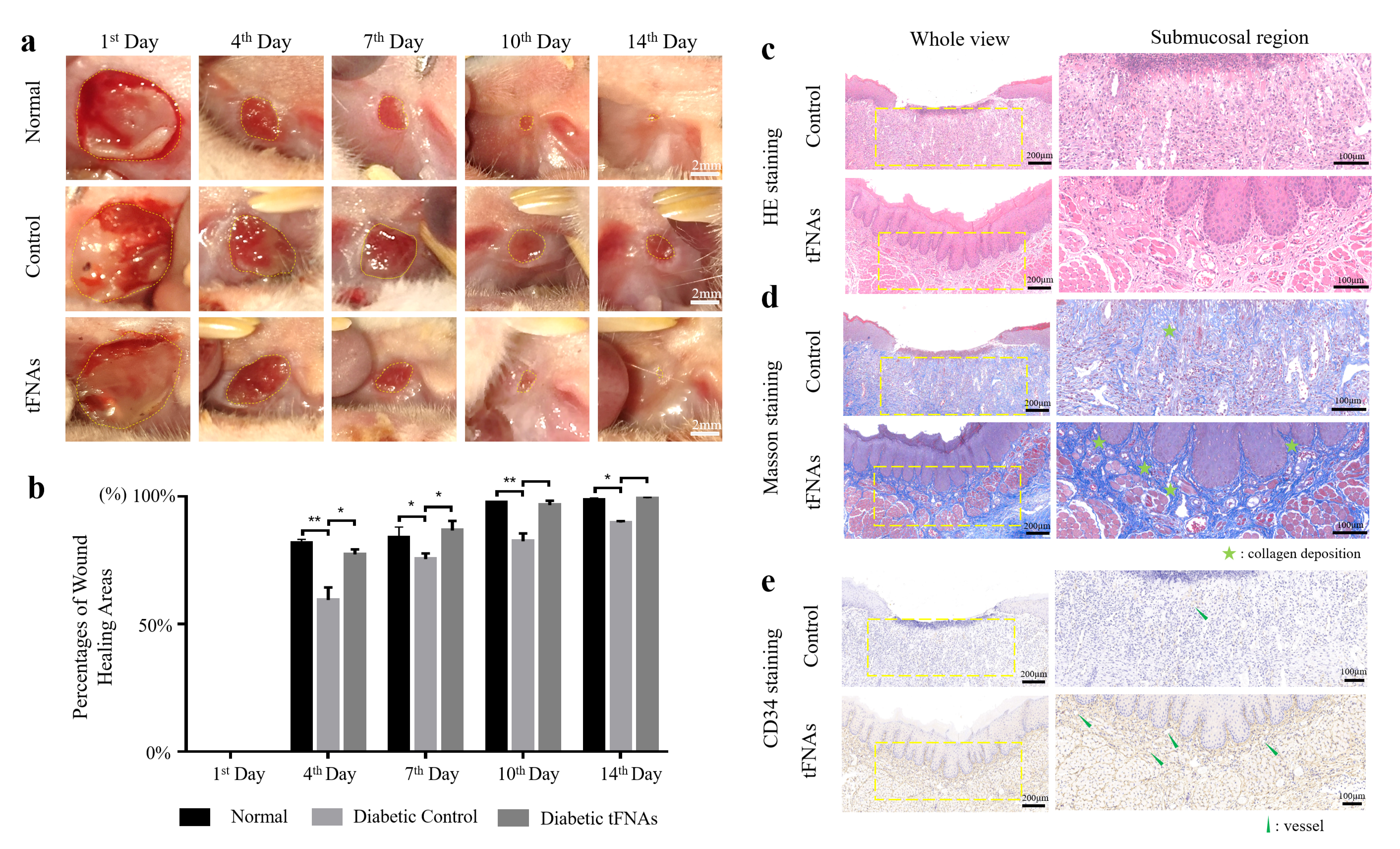

Supplement: Supplementary file 5 — Figure S3 [file 41368_2022_199_MOESM5_ESM.jpg]
