# Supplementary material for: Advances in regenerative medicine applications of tetrahedral framework nucleic acid-based nanomaterials: an expert consensus recommendation
Source: Int J Oral Sci. 2022 Oct 31;14:51. doi: 10.1038/s41368-022-00199-9 (PMC9622686; doi:10.1038/s41368-022-00199-9)
Supplement: Supplementary file 7 — Copyright file of Fig 6 [file 41368_2022_199_MOESM7_ESM.pdf]

Help

Email Support

RightsLink®

SPRINGER NATURE

Tetrahedral framework nucleic acids promote scarless healing of cutaneous wounds via the AKT-signaling pathway

Author: Junyao Zhu et al

Publication: Signal Transduction and Targeted Therapy

Publisher: Springer Nature

Date: Jul 17, 2020

Copyright © 2020, The Author(s)

Creative Commons

This is an open access article distributed under the terms of the Creative Commons CC BY license, which permits unrestricted use, distribution, and reproduction in any medium, provided the original work is properly cited.

You are not required to obtain permission to reuse this article.

To request permission for a type of use not listed, please contact Springer Nature

© 2022 Copyright - All Rights Reserved | Copyright Clearance Center, Inc. | Privacy statement | Terms and Conditions

Comments? We would like to hear from you. E-mail us at customercare@copyright.com
