# Supplementary material for: Advances in regenerative medicine applications of tetrahedral framework nucleic acid-based nanomaterials: an expert consensus recommendation
Source: Int J Oral Sci. 2022 Oct 31;14:51. doi: 10.1038/s41368-022-00199-9 (PMC9622686; doi:10.1038/s41368-022-00199-9)
Supplement: Supplementary file 8 — Copyright file of Fig 7 [file 41368_2022_199_MOESM8_ESM.pdf]

CCC RightsLink®

? Help
Email Support

## Multi-targeted Antisense Oligonucleotide Delivery by a Framework Nucleic Acid for Inhibiting Biofilm Formation and Virulence

SPRINGER NATURE

**Author:** Yuxin Zhang et al

**Publication:** Nano-Micro Letters

**Publisher:** Springer Nature

**Date:** Mar 17, 2020

*Copyright © 2020, The Author(s)*

### Creative Commons

This is an open access article distributed under the terms of the Creative Commons CC BY license, which permits unrestricted use, distribution, and reproduction in any medium, provided the original work is properly cited.

You are not required to obtain permission to reuse this article.
To request permission for a type of use not listed, please contact Springer Nature

© 2022 Copyright - All Rights Reserved | Copyright Clearance Center, Inc. | Privacy statement | Terms and Conditions
Comments? We would like to hear from you. E-mail us at customercare@copyright.com
